# Repulsive interactions instruct synaptic partner matching in an olfactory circuit

**DOI:** 10.21203/rs.3.rs-6099208/v1

**Published:** 2025-03-14

**Authors:** Zhuoran Li, Cheng Lyu, Chuanyun Xu, Ying Hu, David J. Luginbuhl, Asaf B. Lehovic, Jessica M. Priest, Engin Özkan, Liqun Luo

**Affiliations:** 1 Department of Biology and Howard Hughes Medical Institute, Stanford University, Stanford, CA 94305, USA; 2 Biology Graduate Program, Stanford University, Stanford, CA 94305, USA; 3 Department of Biochemistry and Molecular Biology, The Neuroscience Institute and Institute for Biophysical Dynamics, The University of Chicago, Chicago, IL 60637, USA; 4 These authors contributed equally

## Abstract

Neurons exhibit extraordinary precision in selecting synaptic partners. Whereas cell-surface proteins (CSPs) mediating attractive interactions between developing axons and dendrites have been shown to instruct synaptic partner matching^1,2^, it is less clear the degree to which repulsive interactions play a role. Here, using a genetic screen guided by single cell transcriptomes^3,4^, we identified three CSP pairs—Toll2–Ptp10D, Fili–Kek1, and Hbs/Sns–Kirre—in mediating repulsive interactions between non-partner olfactory receptor neuron (ORN) axons and projection neuron (PN) dendrites in the developing *Drosophila* olfactory circuit. Each CSP pair exhibits inverse expression patterns in the select PN-ORN partners. Loss of each CSP in ORNs led to similar synaptic partner matching deficits as the loss of its partner CSP in PNs, and mistargeting phenotypes caused by overexpressing one CSP could be suppressed by loss of its partner CSP. Each CSP pair is also differentially expressed in other brain regions. Together, our data reveal that multiple repulsive CSP pairs work together to ensure precise synaptic partner matching during development by preventing neurons from forming connections with non-cognate partners.

## Main text

A fundamental question in neural development is how the vast number of neurons precisely select their synaptic partners to form functional circuits. The wiring of a neural circuit usually involves multiple coordinated developmental steps: axon guidance to target zones, dendrite patterning, and synaptic partner selection followed by synaptogenesis^5–7^. Even though axon guidance and dendrite patterning can greatly reduce the number of potential partners a neuron encounters at a given time and region^8^, a developing axon must select specific partners among multiple nearby non-partners^9^. The mechanisms by which neural systems reduce multiple candidate synaptic partners to a specific one remain poorly understood.

It is well established that axon guidance involves both attraction towards the target region and repulsion away from non-target regions^10,11^. Besides axon guidance^12–18^, repulsion mediated by cell-surface proteins (CSPs) is used in establishing topographic maps^19,20^, subregion target selection^21^, and dendritic and axonal self-avoidance ^22–28^. However, most known CSPs that instruct the final steps of synaptic partner selection act through attraction. These include homophilic attraction of Teneurins (Ten-m and Ten-a) in *Drosophila* olfactory and neuromuscular systems^29,30^, heterophilic attractions among members of Dpr-DIP family of immunoglobulin superfamily of CSPs in the *Drosophila* optic lobe^31–33^ and neuromuscular system^34^, and homophilic attraction mediated by immunoglobulin^35^ or cadherin^36,37^ families of CSPs in the vertebrate retina. The few repulsion examples include synaptic partner matching in the *Drosophila* neuromuscular system controlled by Wnt4 from non-target muscles^38^ and in the *Drosophila* olfactory system by Fish-lips (Fili) from non-synaptic partners^39^. It remains to be examined how general repulsion is employed as a guiding force in synaptic partner matching.

In the *Drosophila* olfactory circuit, axons of about 50 types of olfactory receptor neurons (ORNs) form one-to-one precise synaptic connections with dendrites of 50 types of projection neurons (PNs) in 50 glomeruli in the antennal lobe^40–42^. During development, PN dendrites coarsely pattern the antennal lobe first^43,44^. For ORN axons, after extending across the antennal lobe prepatterned by PN dendrites, each ORN axon first sends multiple transient branches along its trajectory; those branches that contact partner PN dendrites are then stabilized and undergo further branches, whereas the rest retract^45,46^. Since synaptic partner matching involves retraction of transient ORN axon branches in contact with non-partner PNs, we aimed to identify repulsive CSPs that might function to prevent the formation of misconnections between non-partner PNs and ORNs.

### Inverse expression of three CSP pairs

**Fig. 1 |:**
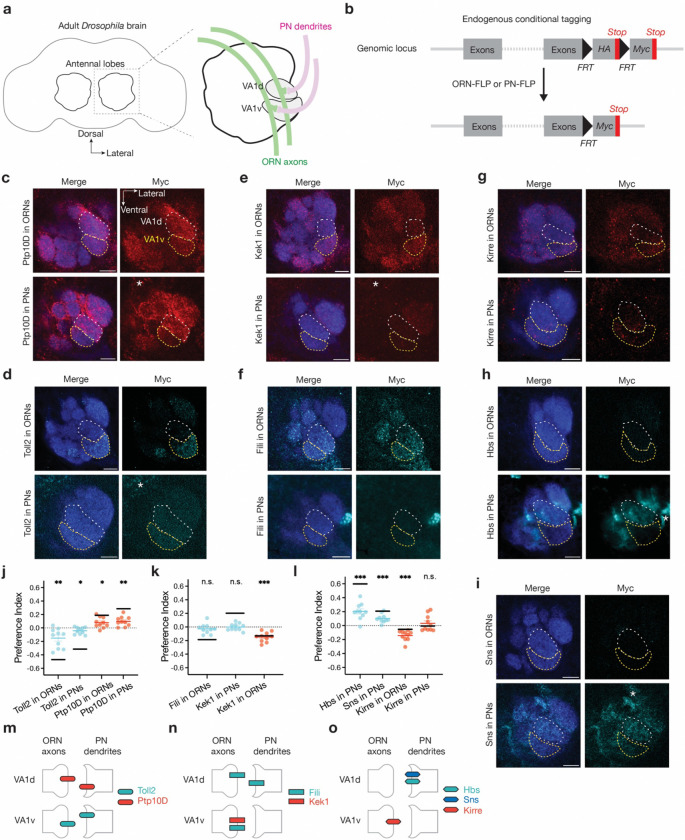
Inverse expression of three CSP pairs in the VA1d and VA1v glomeruli. **a,** Adult *Drosophila* brain and the antennal lobe schematics. Axons of VA1d-ORNs and VA1v-ORNs (green) match with dendrites of VA1d-PNs and VA1v-PNs (magenta), respectively. **b,** Schematic of conditional tagging of CSPs to reveal their endogenous protein expression pattern in whole brain (top) or in specific cell types (bottom), respectively, before and after FLP-mediated recombination. **c–i,** Confocal images showing neuropil staining by N-cadherin antibody (blue) and Myc staining of tagged endogenous CSPs (cyan in **e, g, i, j** and red in **d, f, h**) using ORN-specific *eyFLP* (top tow) or PN-specific *VT033006>FLP* (bottom row). The VA1d (white dotted lines) and VA1v (yellow dotted lines) glomeruli are outlined based on N-cadherin staining. **j–l,** Quantification of the preference index (Methods) of RNA (black horizontal lines) and protein (colored data points) expression levels in ORNs and PNs that innervate the VA1d versus VA1v glomerulus. Preference index > 0 means that expression level in VA1d is higher than in VA1v. Preference index for proteins is calculated based on Myc staining intensity at 42–48h APF (data in **c–i**). For all genotypes, n ≥ 10. Cyan or red lines indicate geometric mean. One-sample t-test comparing to zero. Preference index for mRNAs is calculated based on average expression levels in VA1d-ORN/PNs versus VA1v-ORN/PNs based on the single-cell-transcriptome data at 24–30h APF^3,4^ (black lines), which is consistent with the protein data. **m–o,** Simplified schematic summary of relative CSP expression levels in the VA1d and VA1v PN-ORN pairs during development based on mRNA and protein data (**j–l**; see Extended Data Fig. 2 for a discussion of caveats of each). If the expression level in VA1d- and VA1v- ORNs/PNs is significantly different, we only drew the bars in the cell types with higher expression level as a simplification. We did not draw the Kirre expression level in VA1d- or VA1v-PNs given its preference index is highly variable (**p**) and given that the expression of its partners Hbs and Sns are in ORNs are barely detectable (**i, j**). In this and all subsequent figures, * P < 0.05; ** P < 0.01; *** P < 0.001; n.s. not significant, Scale bars = 10 μm unless otherwise noted.

VA1d and VA1v are neighboring glomeruli that process distinct pheromones^47,48^. Known homophilic attraction molecules mediating matching between synaptic partners, Ten-m and Ten-a, cannot distinguish VA1d-PNs and VA1d-ORNs from VA1v-PNs and VA1v-ORNs, as they all express Ten-m at high level and Ten-a at low level^29^. We hypothesized that additional CSPs should be differentially expressed and instruct synaptic partner matching in these adjacent glomeruli. To identify such CSPs, we performed a genetic screen focusing on PN-ORN matching in the VA1d and VA1v glomeruli (Fig. 1a). We first analyzed the existing single-cell transcriptome data for developing PNs and ORNs^3,4^ at 24–30h after puparium formation (APF), shortly before matching between ORN axons and PN dendrites occurs. We focused on CSPs (including both transmembrane and secreted proteins^49^) that are differentially expressed in VA1d-PNs and VA1v-PNs, or VA1d-ORNs and VA1v-ORNs. We narrowed down to 36 candidate genes with assistance from existing literature, for example, the list of top 100 CSPs enriched in developing antennal lobes revealed by proteomic profiling^50^. We then performed tissue-specific RNA interference (RNAi) against candidate genes selectively in PNs, ORNs, and/or all neurons.

**Extended Data Fig. 1 |:**
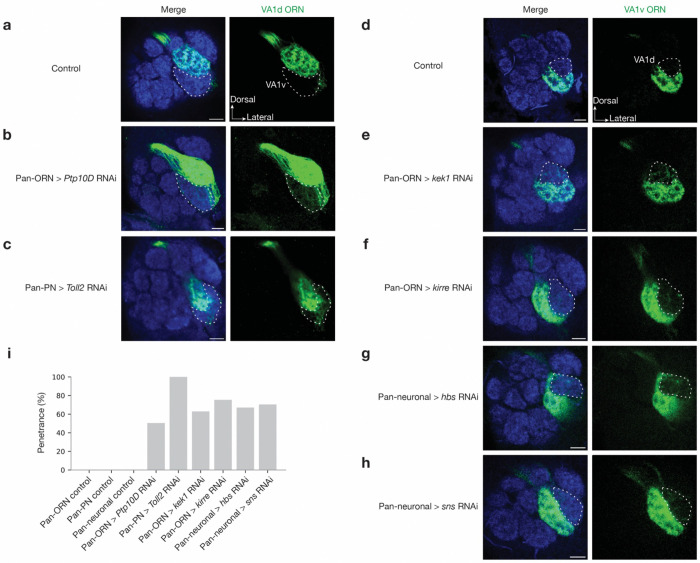
*In vivo* RNAi screen to identify CSPs required for synaptic partner matching. **a–c,** Confocal images of adult antennal lobes showing neuropil staining by N-cadherin antibody (blue) and VA1d-ORN axons (green). The VA1v glomerulus is outlined based on N-cadherin staining. Control VA1d-ORN axons only innervate the VA1d glomerulus dorsal to the VA1v glomerulus (**a**). Some VA1d-ORN axons mistarget to the VA1v glomerulus in *Ptp10D* RNAi expressed from all ORNs (**b**, maximum projection of 3 sections with 1-μm interval), or *Toll2* RNAi expressed in all PNs (**c**). **d–h,** Confocal images of adult antennal lobes showing neuropil staining by N-cadherin antibody (blue) and VA1v-ORN axons (green). The VA1d glomerulus is outlined based on N-cadherin staining. Control VA1v-ORN axons only innervate the VA1v glomerulus ventral to the VA1d glomerulus (**d**). Some VA1v-ORN axons mistarget to the VA1d glomerulus in *kek1* RNAi expressed in all ORNs (**e**), *kirre* RNAi expressed in all ORNs (**f**), *hbs* RNAi expressed in all neurons (**g**), or *sns* RNAi expressed in all neurons (**h**). **i,** Penetrance of the mistargeting phenotypes in **a–h**. For all genotypes, n ≥ 7. Scale bars = 10 μm.

**Extended Data Fig. 2 |:**
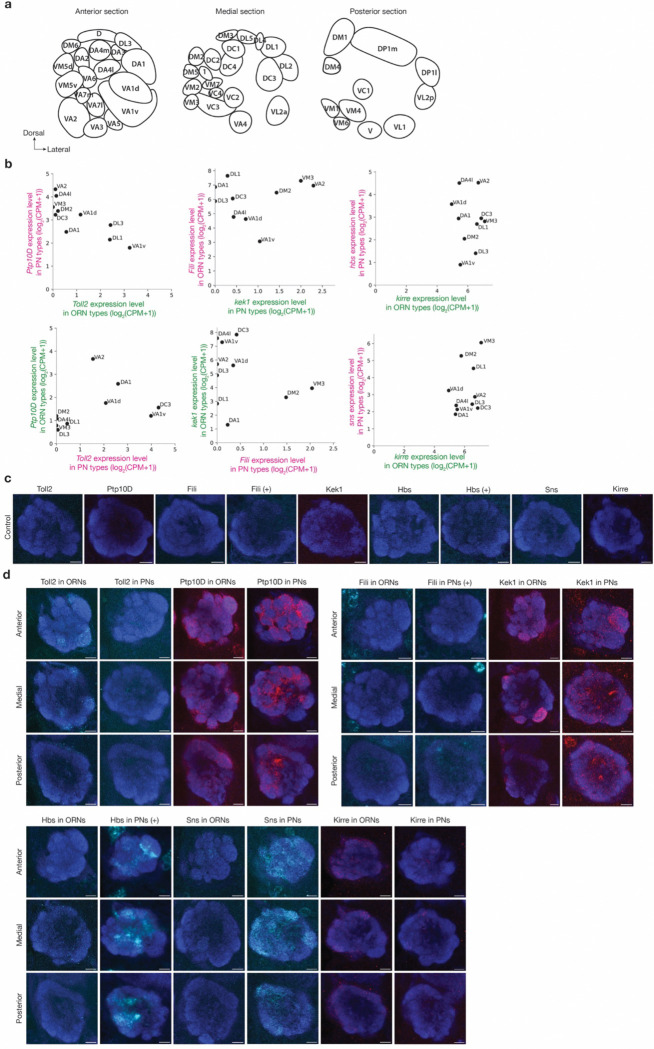
Cell-type-specific expression levels of all CSP pairs across the antennal lobe. **a,** Adult antennal lobe schematic showing glomerular identities in anterior, medial and posterior sections. **b,** CSP pairs’ expression levels in 13 partner PN-ORN pairs with single-cell RNA sequencing data. CPM: counts per million reads. Data adapted from previous published studies^3,4^. **c**, Confocal images showing neuropil staining by N-cadherin antibody (blue) and Myc staining of tagged endogenous CSPs (cyan for Toll2, Fili, Hbs, and Sns; red for Ptp10D, Kek1, and Kirre) without FLP. Without FLP, no Myc signal is detected. Annotation (+) indicate signal amplification for Myc staining. **d,** Confocal images showing neuropil staining by N-cadherin antibody (blue) and Myc staining of tagged endogenous CSPs (cyan for Toll2, Fili, Hbs, and Sns; red for Ptp10D, Kek1, and Kirre) using ORN-specific *eyFLP* or using PN-specific *VT033006>FLP*. The three rows are anterior, medial, and posterior sections of a representative adult brain. Annotation (+) indicate signal amplification for Myc staining. Scale bars = 10 μm. We note that the differential expression patterns of mRNAs and proteins are largely consistent (e.g., Fig. 1j–l). Occasional discrepancies could be caused by (1) post-transcriptional regulations (e.g., protein translation, stability) and (2) different time windows from which mRNA (24–30h APF) and protein (42–48h APF) data were collected. Ideally, protein staining should be done around 30h APF when synaptic partner matching initiates. However, as glomeruli have not formed at that stage, we could not distinguish cell types in which proteins are expressed. 42–48h is the earliest window we could use glomerular identity to infer cell-type-specific expression. It is possible that the expression of some of the CSPs for synaptic partner matching is already downregulated by then.

In wild-type animals, axons of VA1d-ORNs and VA1v-ORNs only innervated the VA1d and VA1v glomeruli, respectively (Extended Data Fig. 1a, d). From the screen, 14 out of 36 candidate genes showed mistargeting phenotypes in either VA1d-ORNs or VA1v-ORNs (Extended Data Fig. 1; Extended Data Table 1; companion manuscript^51^). We note that loss-of-function of any single CSP usually resulted in subtle phenotypes: mistargeting of a small fraction of axons or dendrites (and our single confocal section captured only a small subset of mistargeted axons and dendrites). In the companion manuscript, we showed that only by simultaneously manipulating multiple CSPs in ORNs could we substantially switch their partner PNs^51^. Among these candidate genes, three pairs of CSPs—Toll2–Ptp10D, Fili–Kek1, and Hbs/Sns–Kirre—exhibited largely inverse expression patterns in ORN-PN synaptic partners, particularly in ORNs and PNs that target the VA1d and VA1v glomeruli (Extended Data Fig. 2b). For example, *Toll2* is more highly expressed in VA1v-ORNs than VA1d-ORNs, whereas its partner *Ptp10D* is more highly expressed in VA1d-PNs than VA1v-PNs (Extended Data Fig. 2b, top left panel). Such inverse expression patterns suggest a potential role for these CSP pairs to promote repulsion during synaptic partner matching. We thus focused the rest of our study on these three CSP pairs.

To validate the mRNA-based inverse expression patterns (Extended Data Fig. 2b), we examined the endogenous protein expression levels at 42–48h APF, when glomerular identities first become identifiable (and the matching between partner ORN axons and PN dendrites is mostly complete). To determine cell-type-specific expression patterns, we knocked into the endogenous loci of the three CSP pairs with a modified conditional tag^52,50^ (Fig. 1b). In the absence of the FLP recombinase, these proteins were tagged with HA and no Myc signal was detected in the antennal lobe (Supplementary Video 1–7 and Extended Data Fig. 2c). With ORN-specific FLP or PN-specific FLP, we could visualize endogenous protein expression only in ORNs or PNs by Myc staining (Fig. 1c–i and Extended Data Fig. 2). We found that Toll2 (also named 18-wheeler) and Ptp10D (protein tyrosine phosphatase 10D) were differentially expressed in both PNs and ORNs throughout the antennal lobe (Extended Data Fig. 2d). In the VA1d and VA1v glomeruli, Toll2 exhibited higher expression in VA1v-PNs and VA1v-ORNs, whereas Ptp10D had higher expression in VA1d-PNs and VA1d-ORNs (Fig. 1c, d, j). Using a similar strategy, we found Fili was expressed in both VA1d-ORNs and VA1v-ORNs with no significant differences (Fig. 1f, k). Although Fili’s expression in PNs was undetectable using the conditional tag (Fig. 1f), a previous study showed that Fili exhibits higher expression in VA1d-PNs than VA1v-PNs using immunostaining of Fili antibody and cell-type-specific expression pattern using intersection of ORN- or PN-FLP and *Fili-GAL4*^39^. Kek1 (Kekkon 1) exhibited high expression in VA1v-ORNs than in VA1d-ORNs and low expression in both VA1d-PNs and VA1v-PNs (Fig. 1e, k). For the third CSP pair, Hbs (Hibris) and Sns (Sticks and stones) were minimally expressed in ORNs and exhibited higher expression in VA1d-PNs compared to VA1v-PNs (Fig. 1h, i, l), whereas Kirre (Kin of irre) exhibited higher expression in VA1v-ORNs than VA1d-ORNs (Fig. 1g, l). Using the same preference index to quantify the relative expression in ORNs or PNs that target VA1d vs VA1v, we found that mRNA and protein expression patterns were mostly consistent for all three CSP pairs (Fig. 1j–l).

In summary, based on the relative expression levels of mRNAs and proteins, the Toll2–Ptp10D, Fili–Kek1, and Hbs/Sns–Kirre pairs are expressed in inverse patterns in PN-ORN partners at the VA1d and/or VA1v glomeruli (Fig. 1m–o). Furthermore, all these CSPs are present at the terminals of ORN axons and/or PN dendrites at the nascent glomeruli, consistent with their playing a role in synaptic partner matching.

### Loss of Toll2 or Ptp10D disrupts partner matching

**Fig. 2 |:**
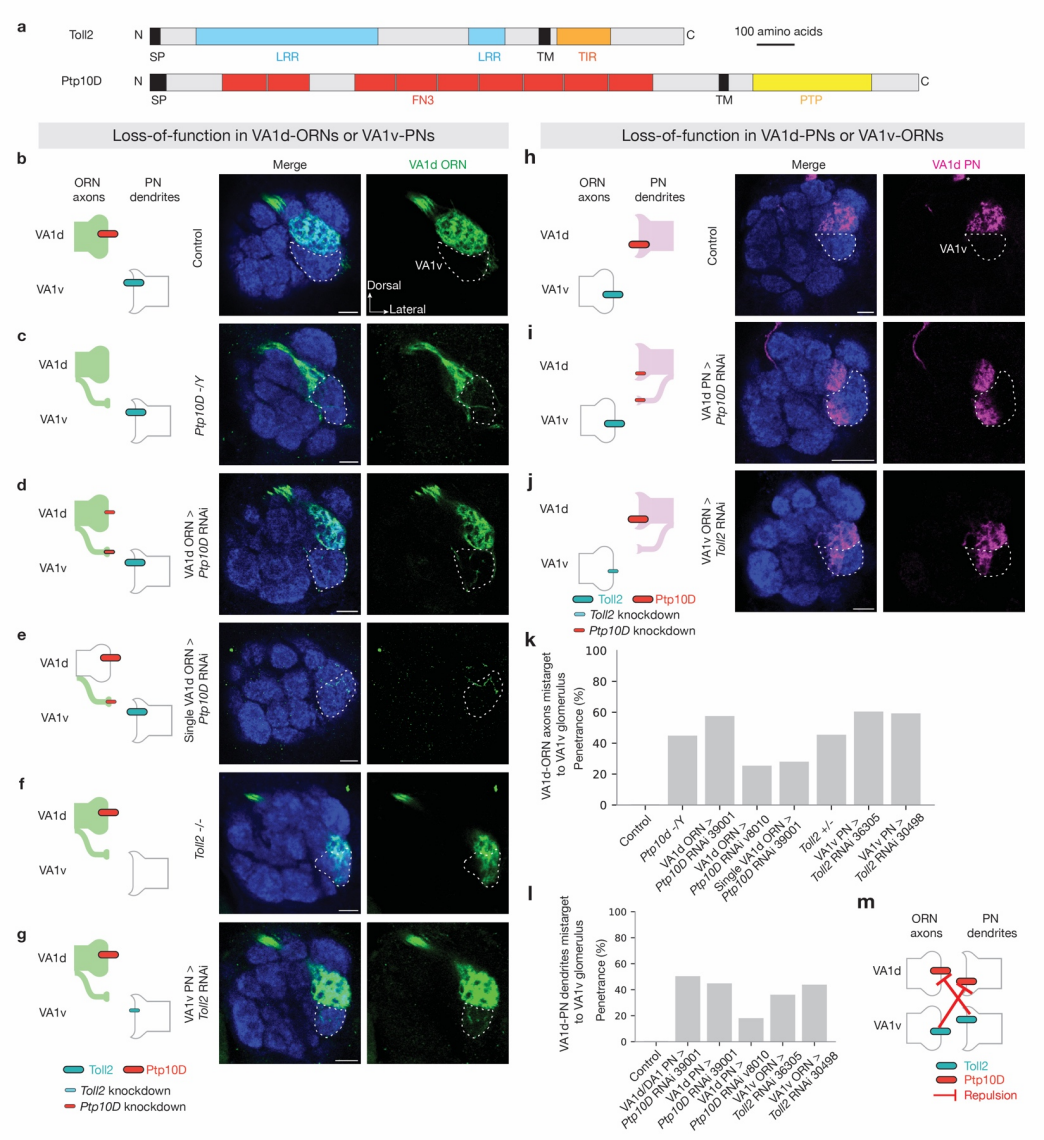
Loss of Ptp10D or Toll2 causes similar mismatching between non-partner PNs and ORNs. **a,** Domain composition of *Drosophila* Toll2 and Ptp10D. TM, transmembrane domain; N, N-terminus; C, C-terminus; SP, signal peptide; LRR, leucine-rich repeat; TIR, Toll/interleukin-1 receptor; FN3, fibronectin type III; PTP, protein tyrosine phosphatase domain. **b–j,** Left column shows experimental schematic (red bar, high Ptp10D expression level; cyan bar, high Toll2 expression level; no bar, knockout; smaller bar, knockdown). Middle and right columns are confocal images of adult antennal lobes showing neuropil staining by N-cadherin antibody (blue) and VA1d-ORN axons (green in **b–g**) or VA1d-PN dendrites (magenta in **h–j**). The VA1v glomerulus is outlined based on N-cadherin staining. Control VA1d-ORN axons innervate the VA1d glomerulus dorsal to the VA1v glomerulus (**b**). Some VA1d-ORN axons mistarget to the VA1v glomerulus in *Ptp10D* hemizygous mutant (**c**), *Ptp10D* RNAi expressed in all (**d**) or single (**e**) VA1d-ORNs, *Toll2* homozygous mutant (**f**), or *Toll2* RNAi expressed in VA1v-PNs (**g**). Control VA1d-PN dendrites only innervate the VA1d glomerulus (**h**). Some VA1d-PN dendrites mistarget to the VA1v glomerulus in *Ptp10D* RNAi expressed in VA1d-PNs (**i**) or *Toll2* RNAi expressed in VA1v-ORNs (**j**). * designates PN cell bodies. Although mistargeting phenotypes are subtle for some genotypes, the penetrance is high. **k, l,** Penetrance of the mistargeting phenotypes in **b–g** (**k**) and **h–j** (**l**). Mistargeting is counted if at least one trackable axon or dendrite innervate inside the VA1v glomerulus based on examining the 3D confocal stacks. For all genotypes, n ≥ 10. **m,** Schematic summary for the function of Ptp10D and Toll2 in VA1d and VA1v glomeruli. —|, repulsive signaling from the open end (sender) to the end with a perpendicular line (receiver).

We first examined the function of Toll2 and Ptp10D in PN-ORN synaptic partner matching. Ptp10D is an evolutionarily conserved single-pass transmembrane protein belonging to the Type III receptor tyrosine phosphatase (RPTP) family (Fig. 2a). Its mammalian orthologs negatively regulate receptor tyrosine kinases (RTKs) through dephosphorylation and play key roles in neurodevelopment^53–55^. In *Drosophila*, Ptp10D is involved in axon guidance at the midline, tracheal tube formation, and cell competition, and was reported to be a receptor of the CSP Sas (Stranded at second)^56–60^. However, single-cell transcriptomic data indicate that Sas is minimally expressed in the antennal lobe^3,4^, suggesting the existence of additional Ptp10D-interacting CSPs.

**Extended Data Fig. 3 |:**
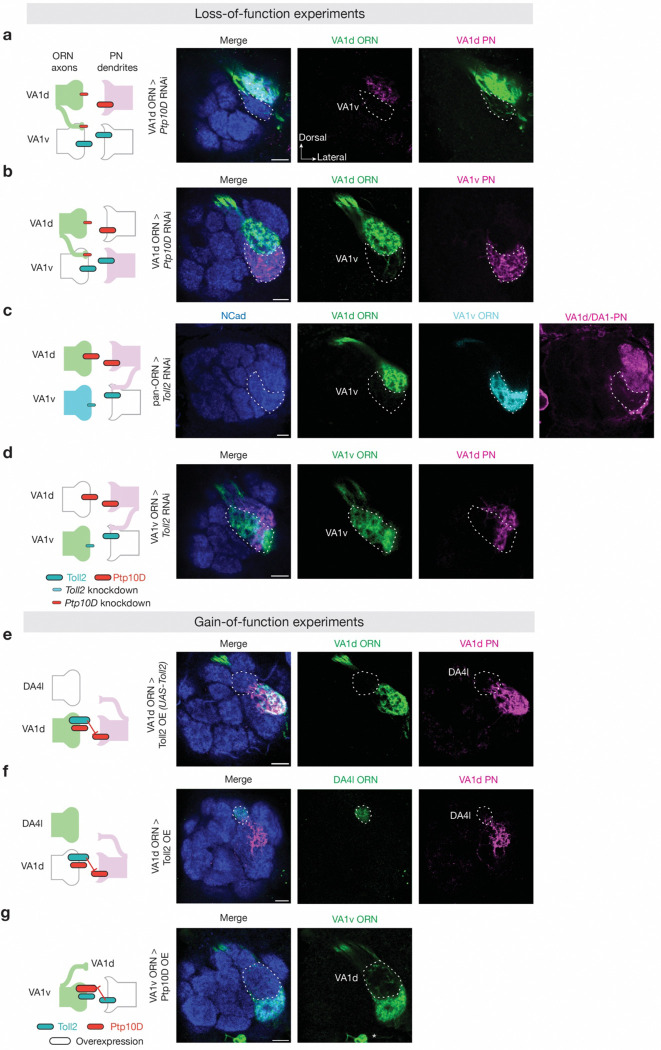
Additional data supporting that Ptp10D and Toll2 mediate PN-ORN repulsion. Schematics on the left column show genetic manipulations (red bar, high expression level of Ptp10D; cyan bar, high expression level of Toll2; smaller bar, knockdown; large bar, overexpression). Right columns are confocal images of representative single sections of adult antennal lobes showing neuropil staining by N-cadherin antibody (blue), and different types of ORN axons (green) or PN dendrites (magenta). Specific glomeruli are outlined based on N-cadherin staining. **a,** Some VA1d-ORN axons (green) mistarget to the VA1v glomerulus in *Ptp10D* RNAi expressed in VA1d-ORNs, whereas VA1d-PN dendrites (magenta) only innervate VA1d glomerulus. This result argues against the possibility that VA1d-ORN axons mistargeting is a secondary effect of VA1d-PN dendrites mistargeting. **b,** Some VA1d-ORN axons (green) mistarget to the VA1v glomerulus and match with VA1v-PN dendrites (magenta) in *Ptp10D* RNAi expressed in VA1d-ORNs. **c,** Some VA1d/DA1-PN dendrites (magenta) mistarget to the VA1v glomerulus and match with VA1v-ORN axons (cyan) in *Toll2* RNAi expressed in all ORNs, whereas VA1d-ORN axons (green) only innervate VA1d glomerulus. This result suggests that Toll2 functions non-cell-autonomously and does not cause repulsion between VA1d-ORNs and VA1v-ORNs. **d,** Some VA1d-PN dendrites (magenta) mistarget to the VA1v glomerulus and match with VA1v-ORN axons (green) in *Toll2* RNAi expressed in VA1v-ORNs. This result suggests that Toll2 functions non-cell-autonomously in VA1v-ORNs to prevent VA1d-PN dendrites to mistarget to the VA1v glomerulus. **e,** Some VA1d-PN dendrites (magenta) mistarget to the DA4l glomerulus following Toll2 overexpression in VA1d-ORNs, whereas VA1d-ORN axons (green) only innervate VA1d glomerulus. This result supports a non-cell-autonomous function for Toll2 and validates the overexpression phenotype seen using a different driver in Fig. 3b. **f,** Some VA1d-PN dendrites (magenta) mistarget to the DA4l glomerulus and match with DA4l-ORN labeled by *Or43a-mCD8GFP* (green) following Toll2 overexpression in VA1d-ORNs. **g,** Some VA1v-ORN axons (green) mistarget to the VA1d glomerulus following Ptp10D overexpression in VA1v-ORNs. Scale bars = 10 μm.

**Extended Data Fig. 4 |:**
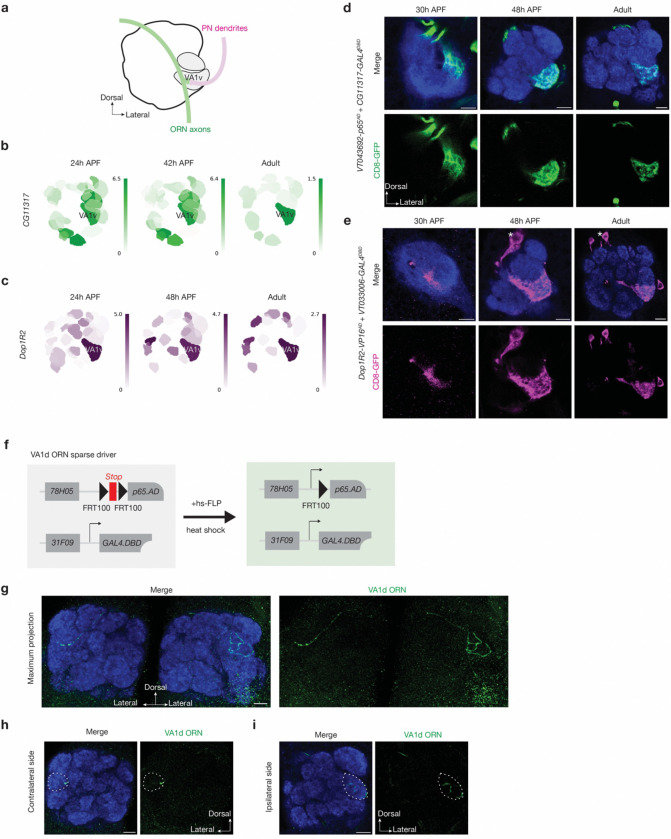
Characterization of the new genetic drivers. **a,** Schematic of VA1v-ORNs and VA1v-PNs matching in the VA1v glomerulus (grey) in adult antennal lobe (black solid line). Green, VA1v-ORNs; Magenta, VA1v-PNs. **b, c,** Single-cell RNA sequencing data showing the expression level of *CG11317* in VA1v-ORNs (**b**) and *Dop1R2* in VA1v-PNs (**c**) are high across developmental stages. Heat map units: log_2_(CPM+1). CPM: counts per million reads. Data adapted from previous published studies^3,4^. **d, e,** Gene-based genetic drivers^70^ express in VA1v-ORNs (green, labeled by a membrane-targeted GFP) (**d**) or VA1v-PNs (magenta, labeled by a membrane-targeted GFP) (**e**) at 30h APF (left), 48h APF (middle) and in adults (right). VA1v glomerulus location is verified by neuropil staining of N-cadherin (NCad) antibody (blue). *, designates PN cell body. **f,** Schematic for VA1d-ORN sparse drivers derived from previously described method^97^. The transcription activation domain (AD) is controlled by the enhancer *GMR78H05* and gated by *FRT100-STOP-FRT100. FRT100* sites are ~1% efficiency as of wild-type FRT sites. STOP represent a transcription termination sequence. Heat-shock-induced FLP expression enables *split-GAL4* expression in a single VA1d-ORN. **g–i,** VA1d-ORN sparse driver enable single VA1d-ORN labeling (green, labeled by a membrane-targeted GFP) as shown in the maximum projection (**g**), single contralateral section (**h**), or single ipsilateral section (**i**). The VA1d glomerulus location is verified by neuropil staining of N-cadherin (NCad) antibody (blue). Scale bars = 10 μm.

To validate the *Ptp10D* RNAi phenotypes from our screen (Extended Data Fig. 1b), we labeled VA1d-ORN axons in *Ptp10D* hemizygous mutant^56^ animals and observed similar phenotype as pan-ORN *Ptp10D* RNAi: VA1d-ORN axons mistargeted to the VA1v glomerulus (Fig. 2c, k). Is this a cell-autonomous effect of VA1d-ORNs or a non-cell-autonomous effect caused by its loss in other ORNs types? Given that VA1d-ORNs exhibited high Ptp10D expression (Fig. 1m), we tested whether Ptp10D is required in VA1d-ORNs for their proper targeting. We knocked down *Ptp10D* specifically in VA1d-ORNs using a VA1d-ORN-specific-GAL4 driver^46^ and observed similar mistargeting of VA1d-ORNs to the VA1v glomerulus and mismatching with VA1v-PN dendrites with multiple RNAi lines (Fig. 2d, k and Extended data Fig. 3a, b). Furthermore, using a sparse VA1d-ORN GAL4 driver^46^ (Extended Data Fig. 4f–i) to knock down *Ptp10D* in single VA1d-ORN also caused axon branches to mistarget to the VA1v glomerulus (Fig. 2e, k), indicating that Ptp10D acts cell-autonomously in VA1d-ORNs to prevent their mismatching with VA1v-PNs.

Based on the inverse expression pattern of Ptp10D and Toll2 (Fig. 1n and Extended Data Fig. 2), we tested whether Toll2 plays a similar role in the precise targeting of VA1d-ORNs. Toll2 is a single-pass transmembrane protein belonging to the Toll-like receptor family, with a leucine-rich repeat (LRRs) in its extracellular domain and a Toll/interleukin-1 receptor (TIR) domain in the intracellular domain (Fig. 2a). Toll2 plays an evolutionarily conserved roles in regulating innate immunity and participating in developmental processes^61–65^. Although other Toll receptors have been shown to instruct neurite targeting or inhibit synaptic initiation^66–69^, whether Toll2 plays a role in neural development has remained unclear. We found that both pan-PN RNAi-mediated knockdown of *Toll2* and *Toll2* mutant^61^ resulted in VA1d-ORN axons mistargeting to the VA1v glomerulus (Fig. 2f, k and Extended Data Fig. 1c).

Given that Toll2 is highly expressed in VA1v-PNs (Fig. 1m), we hypothesized that Toll2 in VA1v-PN dendrites sends a trans-cellular repulsive signal to VA1d-ORN axons to prevent misconnection between them. To manipulate Toll2 specifically in VA1v-PNs, we identified a VA1v-PN driver that labels VA1v-PNs across developmental stages^70^ (Extended Data Fig. 4a–e). Indeed, we found that Toll2 knockdown in VA1v-PNs resulted in VA1d-ORN axons mistargeting to the VA1v glomerulus, phenocopying Ptp10D knockdown in VA1d-ORNs (Fig. 2g, k). Thus, Toll2 acts in VA1v-PNs whereas Ptp10D acts in VA1d-ORNs to prevent misconnections between VA1d-ORN axons and VA1v-PN dendrites (Fig. 2m).

Since Ptp10D and Toll2 were also highly expressed in VA1d-PNs and VA1v-ORNs, respectively (Fig. 1m), we examined whether they are similarly required for preventing mismatching between VA1v-ORNs and VA1d-PNs. We found that cell-type-specific knockdown of Ptp10D in VA1d-PNs and Toll2 in VA1v-ORNs caused similar phenotypes: VA1d-PN dendrites mistargeted to the VA1v glomerulus (Fig. 2h–j, l). Conversely, no mistargeting phenotype was observed in VA1d- or VA1v-ORN axons when Toll2 was knocked down in ORNs (Extended Data Fig. 3c, d), suggesting that Toll2 does not function cell-autonomously in VA1d- or VA1v-ORNs and does not mediate interactions between VA1d-ORNs and VA1v-ORNs. Altogether, these data suggest that both Ptp10D and Toll2 act in both PNs and ORNs to prevent mismatching between non-partners (Fig. 2m).

### Trans-cellular interactions of Toll2 and Ptp10D

**Fig. 3 |:**
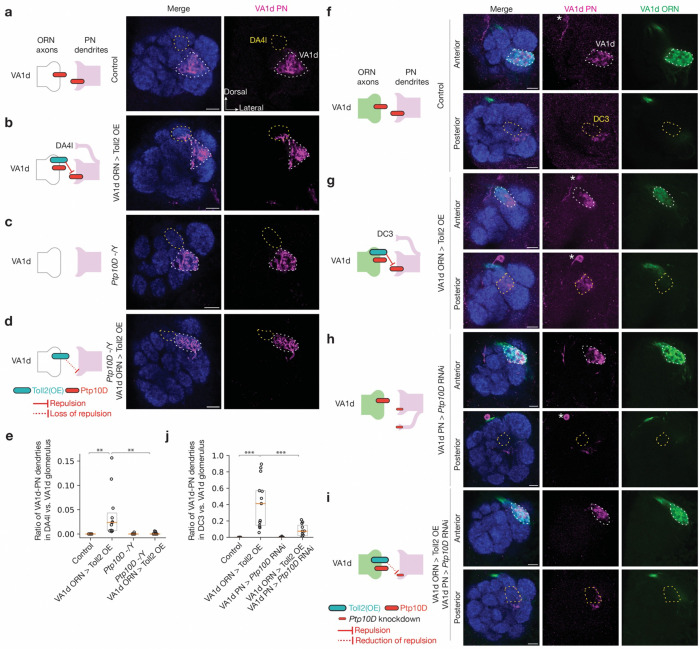
Ptp10D and Toll2 promote trans-cellular repulsive interactions. **a–d,** Left column shows experimental schematic (red bar, high expression level of Ptp10D; large cyan bar, Toll2 overexpression (OE); no bar, knockout). Middle and right columns are confocal images showing neuropil staining by N-cadherin antibody (blue) and VA1d-PN dendrites (magenta). The VA1d (white dashed lines) and DA4l (yellow dashed lines) glomeruli are outlined based on N-cadherin staining. Control VA1d-PN dendrites only innervate the VA1d glomerulus (**a**). Some VA1d-PN dendrites mistarget to the DA4l glomerulus following Toll2 overexpression in VA1d-ORNs (**b**). No VA1d-PN dendrites mistarget to the DA4l glomerulus in *Ptp10D* hemizygous mutant (**c**). Almost no VA1d-PN dendrites mistarget to the DA4l glomerulus when Toll2 overexpression in VA1d-ORNs was performed in *Ptp10D* hemizygous mutant (**d**). **e,** Quantification of mistargeting ratio of VA1d-PN dendrites in the DA4l versus VA1d glomerulus for **a–d**. **f–i,** Left column shows experimental schematic (red bar, high expression level of Ptp10D; large cyan bar, Toll2 overexpression; no bar, knockout; smaller bar, knockdown; red line, repulsion; dotted red line: reduction of repulsion). Right three columns are confocal images showing neuropil staining by N-cadherin antibody (blue), VA1d-PN dendrites (magenta), and VA1d-ORN axons (green). The top and bottom rows are optical sections in the anterior and posterior parts of a representative antennal lobe, respectively. VA1d (white dashed lines) and DC3 (yellow dashed lines) glomeruli are outlined based on N-cadherin staining on the top and bottom row, respectively. In control, VA1d-PN dendrites only innervate the VA1d glomerulus and fully overlap with VA1d-ORN axons (**f**). VA1d-PN dendrites overlap less with VA1d-ORN axons within the VA1d glomerulus and mistarget to the DC3 glomerulus following Toll2 overexpression in VA1d-ORNs (**g**). No VA1d-PN dendrites mistarget to the DC3 glomerulus in *Ptp10D* RNAi expressed in VA1d-PNs (**h**). VA1d-PN dendrites overlap more with VA1d-ORN axons and mistarget less to DC3 glomerulus when Toll2 overexpression in VA1d-ORNs is combined with *Ptp10D* knockdown in VA1d-PNs (**i**). The different mistargeting regions in (**g**) and (**b**) likely result from different Toll2 overexpression levels using different binary systems (Methods). Both DA4l-ORNs and DC3-ORNs express low levels of Ptp10D, consistent with our repulsion model. * designates PN cell bodies. **j,** Quantification of the mistargeting ratio of VA1d-PN dendrites in the DC3 versus VA1d glomerulus. For all genotypes, n ≥ 10. Boxes indicate geometric mean and 25% to 75% range. Kruskal–Wallis test with Bonferroni’s multiple comparison.

We next tested whether Toll2 and Ptp10D work together to prevent mismatching between non-partner PN-ORN via trans-cellular interactions. To do so, we first overexpressed Toll2 specifically in VA1d-ORNs, where the endogenous Toll2 level is low. This caused some of their partner VA1d-PN dendrites to mismatch with DA4l-ORN axons (where Ptp10D level is low) in the DA4l glomeruli, whereas VA1d-ORN axons did not mistarget to other glomeruli (Fig. 3a, b, e and Extended Data Fig. 3e, f). This result supports the repulsion hypothesis: misexpressed Toll2 in VA1d-ORN axons sent a trans-cellular signal to repel the partner VA1d-PN dendrites away from them. Similarly, we overexpressed Ptp10D specifically in VA1v-ORNs whose synaptic partner VA1v-PNs expressed high level of Toll2; this caused VA1v-ORN axons to mistarget to the VA1d glomerulus (Extended Data Fig. 3g).

Next, we tested whether the Toll2 repulsive signal was received by Ptp10D, which was highly expressed in VA1d-PN dendrites. We combined Toll2 overexpression in VA1d-ORNs with loss of Ptp10D. The mistargeting level of VA1d-PN dendrites to the DA4l glomerulus was significantly reduced in *Ptp10D* hemizygous mutant animals (Fig. 3d, e). *Ptp10D* hemizygous itself did not cause VA1d-PN dendrite mistargeting to DA4l, even though some VA1d-PN dendrites mistargeted to VA1v (Fig. 3c, e). This suppression of mistargeting to the DA4l glomerulus suggests that Ptp10D is necessary to mediate the Toll2 overexpression phenotype, and thus Toll2 and Ptp10D function together to mediate repulsion.

As Ptp10D had high expression in both VA1d-PNs and VA1d-ORNs, the experiment above did not distinguish whether the suppression by Ptp10D knockout was a result of *cis-* or *trans*-interaction between Toll2 and Ptp10D, or a result of loss of Ptp10D in other glomeruli besides VA1d. To distinguish between these possibilities, we manipulated Toll2 in VA1d-ORNs and Ptp10D in VA1d-PNs simultaneously using two orthogonal binary expression systems. In wild-type flies, dually labeled VA1d-ORN axons and VA1d-PN dendrites largely intermingled with each other (Fig. 3f, j). Overexpressed Toll2 in VA1d-ORNs caused VA1d-PN dendrites to segregate from VA1d-ORN axons within the VA1d glomerulus or mistarget to the nearby DC3 glomerulus (Fig. 3g, j). Simultaneous overexpression of Toll2 in VA1d-ORNs and knockdown of Ptp10D in VA1v-PNs suppressed the VA1d-PN dendrite phenotypes caused by Toll2 overexpression alone (Fig. 3i, j), whereas Ptp10D knockdown in VA1d-PNs alone did not cause a similar phenotype (Fig. 3h, j). Taken together with the inverse expression of Toll2 and Ptp10D and their similar loss-of-function phenotypes, these trans-cellular interaction data support a model in which Toll2 sends and Ptp10D receives a repulsive signal to prevent matching between non-partner ORNs and PNs (Fig. 2m).

### Fili and Kek1 for non-partner PN-ORN repulsion

**Fig. 4 |:**
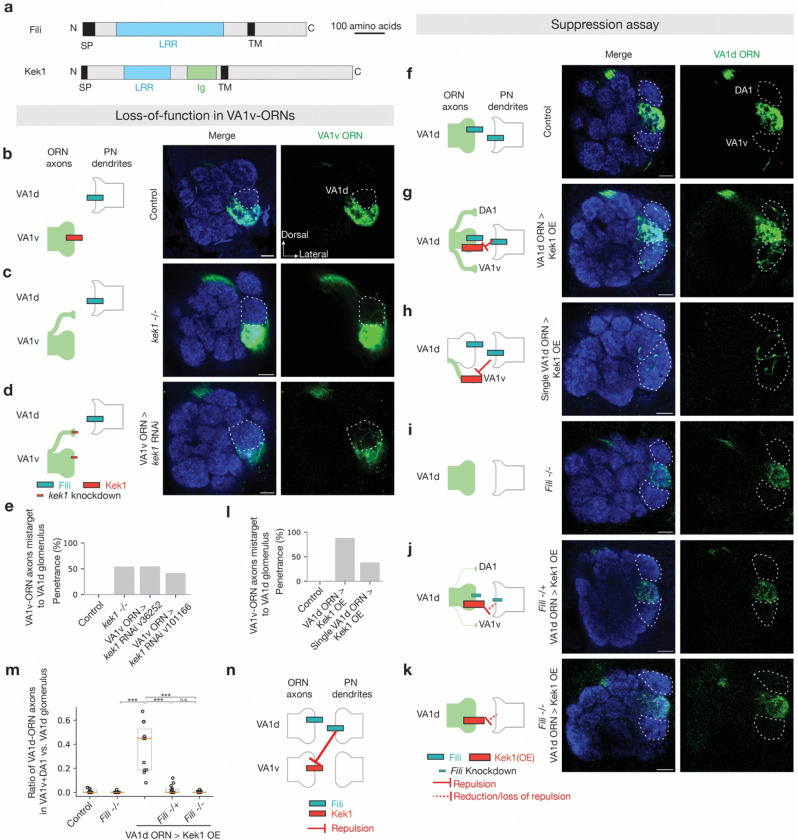
Repulsive interactions between Fili and Kek1. **a,** Domain composition of *Drosophila* CSPs Fili and Kek1. TM, transmembrane domain; N, N-terminus; C, C-terminus; SP, signal peptide; LRR, leucine-rich repeat; Ig, immunoglobulin. **b–d,** Left column shows experimental schematic (red bar, high expression level of Kek1; cyan bar, high expression level of Fili; no bar, knockout; smaller bar, knockdown). Middle and right columns are confocal images showing adult antennal lobe neuropil staining by N-cadherin antibody (blue) and VA1v-ORN axons (green). The VA1d glomerulus is outlined based on N-cadherin staining. Control VA1v-ORN axons only innervate the VA1v glomerulus ventral to the VA1d glomerulus (**b**). Some VA1v-ORN axons mistarget to the VA1d glomerulus in *kek1* mutant (**c**) or *kek1* RNAi expressed in VA1v-ORNs (**d**). **e,** Penetrance of the mistargeting phenotypes in **b–d**. For all genotypes, n ≥ 10. **f–k,** Left column shows experimental schematic (large red bar, Kek1 overexpression (OE); cyan bar, high expression level of Fili; no bar, knockout; smaller bar, heterozygous knockout). Middle and right columns are confocal images showing neuropil staining by N-cadherin antibody (blue) and VA1d-ORN axons (green). DA1 and VA1v glomeruli are outlined based on N-cadherin staining. Control VA1d-ORN axons only innervate the VA1d glomerulus ventral to the DA1 glomerulus and dorsal to the VA1v glomerulus (**f**). Some VA1d-ORN axons mistarget to the DA1 and VA1v glomeruli following Kek1 overexpression in all (**g**) or single (**h**) VA1d-ORNs. No VA1d-ORN axons mistarget to the DA1 or VA1v glomeruli in *Fili* mutant (**i**). Almost no VA1d-ORN axons mistarget to the DA1 and VA1v glomeruli when Kek1 overexpression in VA1d-ORNs was performed in *Fili* heterozygous (**j**) or homozygous (**k**) mutant. **l,** Penetrance of the mistargeting phenotypes in **f–h**. For all genotypes, n ≥ 10. **m,** Quantification of the mistargeting ratio of VA1d-ORN axons in the DA1 and VA1v versus VA1d glomerulus. For all genotypes, n ≥ 10. Boxes indicate geometric mean and 25% to 75% range. Kruskal–Wallis test with Bonferroni’s multiple comparison. **n,** Schematic summary for the function of Kek1 and Fili in the VA1d and VA1v glomeruli. **—|**, repulsive signaling from the open end (sender) to the end with a perpendicular line (receiver).

To study the function of the other CSP pairs, we performed similar loss-of-function and suppression experiments as with Toll2–Ptp10D. A previous study showed that Fili is required in VA1d/DC3-PNs to prevent mistargeting of VA1v-ORN axons to the VA1d glomerulus^39^. Yet Fili’s CSP partner remained elusive. We found that Kek1 was a top candidate based on the inverse expression pattern with Fili and the mistargeting phenotype of VA1v-ORN axons resulting from pan-ORN knockdown of Kek1 (Fig. 1n, Extended Data Fig. 1e, and Extended Data Fig. 2). Kek1 and Fili both contain leucine-rich repeat (LRRs) in their extracellular domain (Fig. 4a). During *Drosophila* eye development, Kek1 inhibits epidermal growth factor receptor (EGFR) activity through the LRR domain^71,72^. In the developing CNS in *Drosophila*, Kek1 is expressed in restricted patterns, but its function in neural development is poorly understood^73^. We found that both homozygous mutant of *kek1*^74^ and *kek1* knockdown in VA1v-ORNs caused VA1v-ORN axons to mistarget to the VA1d glomerulus (Fig. 4b–e), phenocopying the loss of Fili in VA1d/DC3 PNs^39^.

To further investigate whether Fili and Kek1 work together to prevent misconnections between non-partner PN-ORN, we overexpressed Kek1 specifically in VA1d-ORNs, whose synaptic partner VA1d-PNs expressed a relatively high level of Fili^39^. This caused VA1d-ORN axons to mistarget to the neighboring VA1v and DA1 glomeruli (Fig. 4f, g, m), whose PN dendrites mostly do not express Fili (Fili appears to be expressed in a small portion of DA1-PN dendrites neighboring the VA1d glomerulus)^39^. To test whether the Kek1 overexpression phenotype was caused by its interaction with Fili, we overexpressed Kek1 in *Fili* mutant^39^ animals (Fig. 4i, m) and found the overexpression phenotype was much less severe in *Fili* heterozygous mutant (Fig. 4j, m) and was nearly fully suppressed in *Fili* homozygous mutant (Fig. 4k, m). In addition, overexpressing Kek1 in single VA1d-ORN using the VA1d sparse driver produced a similar mistargeting phenotype (Fig. 4h, l), indicating that Kek1 acts cell-autonomously. Although VA1d-ORNs and VA1v-ORNs also expressed Fili (Fig. 1n), a previous study showed that Fili knockout in ORNs does not cause any mistargeting phenotype of VA1d- and VA1v-ORN axons^39^. Together, these data suggest that Fili and Kek1 work together to prevent the misconnections between non-partner PN-ORN, with Fili sending and Kek1 receiving the trans-cellular repulsive signal (Fig. 4n).

### Hbs/Sns and Kirre for non-partner PN-ORN repulsion

**Fig. 5 |:**
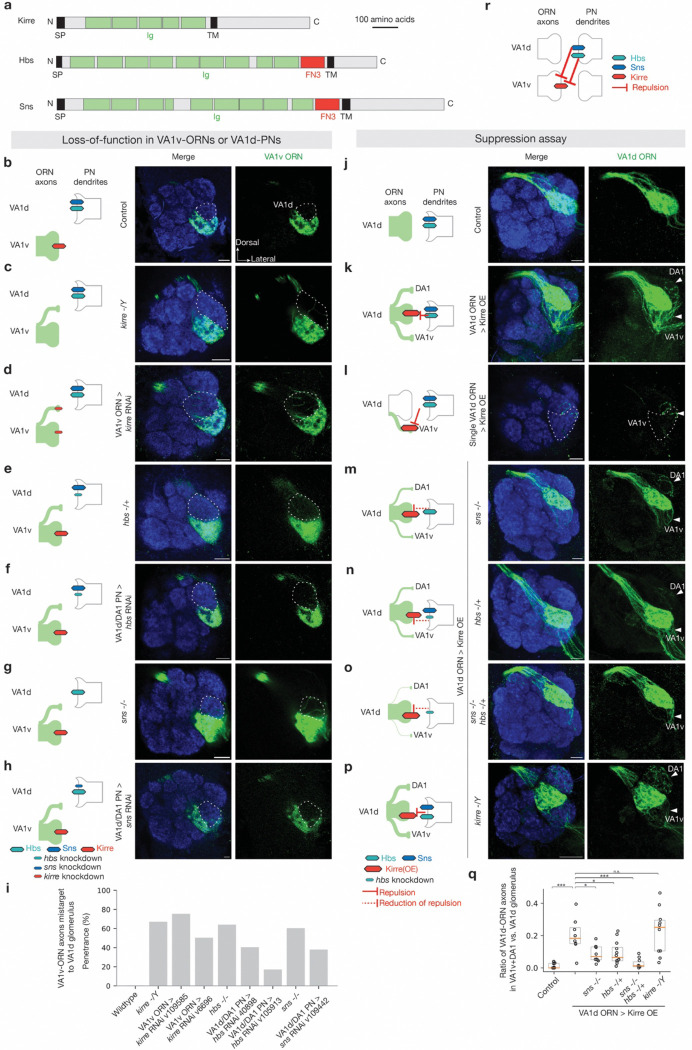
Repulsive interactions between Kirre and Hbs/Sns. **a,** Domain composition of *Drosophila* cell-surface proteins Kirre, Hbs, and Sns. TM, transmembrane domain; N, N-terminus; C, C-terminus; SP, signal peptide; Ig, immunoglobulin; FN3, fibronectin type III. **b–h,** Left column shows experimental schematic (red bar, high expression level of Kirre; cyan bar, high expression level of Hbs; blue bar, high expression level of Sns; no bar, knockout; smaller bar, knockdown). Middle and right columns are confocal images of adult antennal lobes showing neuropil staining by N-cadherin antibody (blue) and VA1v-ORN axons (green). The VA1d glomerulus is outlined based on N-cadherin staining. Control VA1v-ORN axons only innervate the VA1v glomerulus ventral to the VA1d glomerulus (**b**). Some VA1v-ORN axons mistarget to the VA1d glomerulus in *kirre* hemizygous mutant (**c**), *kirre* RNAi expressed in VA1v-ORNs (**d**), *hbs* heterozygous mutant (**e**), *hbs* RNAi expressed in DA1-PNs and VA1d-PNs (**f**), *sns* homozygous mutant (**g**), or *sns* RNAi expressed in DA1-PNs and VA1d-PNs (**h**). **i,** Penetrance of the mistargeting phenotypes in **b–h**. For all genotypes, n ≥ 10. **j–p,** Left column shows experimental schematic (large red bar, Kirre overexpression (OE); cyan bar, high expression level of Hbs; blue bar, high expression level of Sns; no bar, knockout; smaller bar, heterozygous knockout). Middle and right columns are maximum projections (**j–k, m–p**) or a single section (**l**) of confocal images showing neuropil staining by N-cadherin antibody (blue) and VA1d-ORN axons (green). Arrowheads indicate the DA1 and VA1v glomeruli based on N-cadherin staining. Control VA1d-ORN axons only innervate the VA1d glomerulus ventral to the DA1 glomerulus and dorsal to the VA1v glomerulus (**j**). Some VA1d-ORN axons mistarget to the DA1 and VA1v glomeruli following Kirre overexpression in all (**k**) or single (**l**) VA1d-ORNs. Comparing to Kirre overexpression alone, fewer VA1d-ORN axons mistarget to the DA1 and VA1v glomeruli when Kirre overexpression in VA1d-ORNs was performed in *sns* homozygous mutant (**m**), *hbs* heterozygous mutant (**n**), or the combination of them (**o**). VA1d-ORN axons still mistarget to the DA1 and VA1v glomerulus when Kirre overexpression in VA1d-ORNs was performed in *kirre* hemizygous mutant (**p**). **q,** Quantification of the mistargeting ratio of VA1d-ORN axons in the DA1 and VA1v versus VA1d glomerulus. For all genotypes, n ≥ 10. Boxes indicate geometric mean and 25% to 75% range. Kruskal–Wallis test with Bonferroni’s multiple comparison. **r,** Schematic summary for the function of Kirre, Hbs, and Sns in ORN-PN matching in the VA1d and VA1v glomeruli. **—|**, repulsive signaling from the open end (sender) to the end with a perpendicular line (receiver).

Hbs/Sns and Kirre are evolutionarily conserved immunoglobulin family of ligand-receptor pairs with conserved binding sites^75^ (Fig. 5a). In *Drosophila*, Hbs/Sns and Kirre were first reported to function in myoblast fusion^76–78^, and were later found to be required for nephrocytes functions^79^ and proper neural circuit wiring^80^. In the wiring of the *Drosophlia* olfactory system, the expression patterns and mistargeting phenotypes we observed raised the possibility that Hbs/Sns and Kirre might mediate repulsive interaction (Fig. 1o). We found that *kirre* mutant^79^, *hbs* mutant^77^, and *sns* mutant^76^ recapitulated the RNAi phenotype of VA1v-ORN axons mistargeting to the VA1d glomerulus (Fig. 5b, c, e, g, i, and Extended Data Fig. 1f–h). Furthermore, VA1v-ORN-specific knockdown of Kirre, as well as the VA1d-PN-specific knockdown of Hbs and Sns, all caused a similar phenotype: mistargeting of VA1v-ORNs to the VA1d glomerulus (Fig. 5b, d, f, h, i). Thus, Kirre, Hbs, Sns are all required to prevent VA1v-ORNs from matching with VA1d-PNs, with Kirre acting in VA1v-ORNs while Hbs/Sns acting in VA1d-PNs.

**Extended Data Fig. 5 |:**
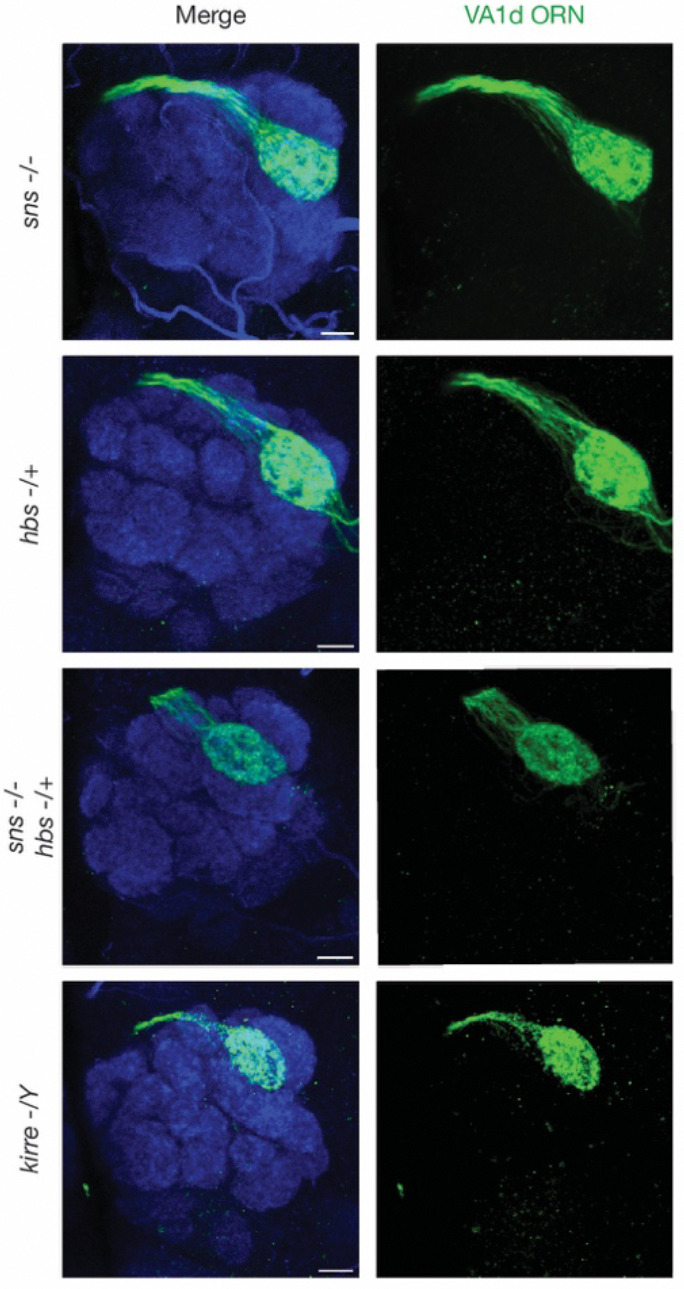
VA1d-ORN axons target normally in *sns*, *hbs*, or *kirre* mutant animals. Confocal images of adult antennal lobes showing neuropil staining by N-cadherin antibody (blue) and VA1d-ORN axons (green). VA1d-ORN axons only innervate the VA1d glomerulus dorsal to the VA1v glomerulus in *sns* homozygous mutant, *hbs* heterozygous mutant, *hbs*/*sns* double mutant, or *kirre* hemizygous mutant. Scale bars = 10 μm.

To examine whether Hbs/Sns and Kirre work together to instruct synaptic partner matching, we performed genetic interaction experiments. We first overexpressed Kirre in VA1d-ORNs, whose partner, VA1d-PNs, highly expresses Hbs and Sns (Fig. 1o). Kirre overexpression in all VA1d-ORNs or single VA1d-ORN led to mistargeting of some VA1d-ORN axons into VA1v and DA1 glomeruli (Fig. 5j–l, q), whose PNs have low levels of Hbs and Sns, suggesting cell-autonomous function of Kirre as a repulsive receptor. Overexpressing Kirre in *hbs* mutant, *sns* mutant, or *hbs*/*sns* double mutant^77^ background reduced mistargeting phenotypes for VA1d-ORN axons (Fig. 5m–o, q). None of the mutants alone had any VA1d-ORNs mistargeting phenotype in the absence of Kirre overexpression (Extended Data Fig. 5). Since Kirre can also mediate homophilic binding^75^, we tested whether Kirre homophilic attraction is responsible for the mistargeting phenotype we observed. We found that overexpressing Kirre specifically in VA1d-ORNs in *kirre* hemizygous mutant animals did not change the overexpression phenotype (Fig. 5p, q), arguing against a contribution for trans-cellular Kirre homophilic interaction in synaptic partner matching. Together, these data support that heterophilic repulsion between Hbs/Sns as the ligands and Kirre as the receptor prevents VA1v-ORNs from mistargeting to the VA1d glomerulus (Fig. 5r).

**Extended data Fig. 6 |:**
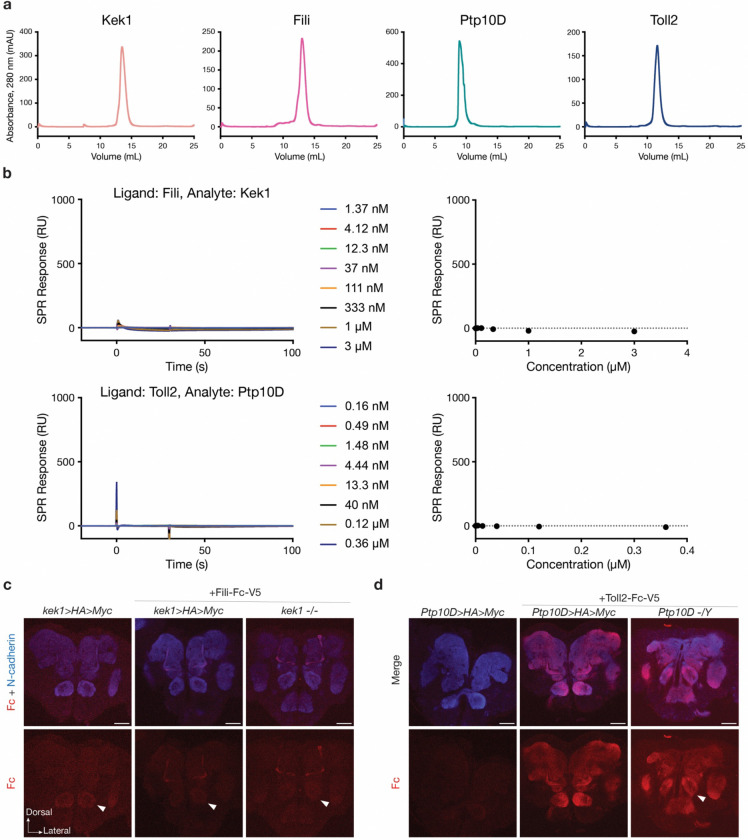
Biochemical assays to test direct binding between CSP pairs. **a,** Size-exclusion chromatography curves for four extracellular domains (ectodomains) purified on a Superdex 200 Increase 10/300 column on an ÄKTA Pure system, reporting absorbance at 280 nm with a path length of 0.2 cm. **b,** Surface plasmon resonance sensorgams of 30 s injections (left) and analyte concentration vs. response plots (right) show no direct physical interaction between Fili and Kek1, and between Toll2 and Ptp10D. **c,** Confocal images of pupal brains (48h APF) showing neuropil staining by N-cadherin antibody (blue), and binding of Fili-Fc-V5 in live pupal brains followed by fixing and staining against Fc (red). *kek1* conditionally tagged brains (middle column) incubated with Fili-Fc-V5 proteins in medium do not exhibit an increase of Fc signal in the antennal lobe (arrowheads) comparing to the control without the addition of Fili-Fc-V5 (left column) or Fili-Fc-V5 in *kek1* homozygous mutant brain (right column). **d,** Confocal images of pupal brains (48h APF) showing neuropil staining by N-cadherin antibody (blue), and binding of Toll2-Fc-V5 in live pupa brains followed by fixing and staining against Fc (red). Control *Ptp10D* conditionally tagged brains incubated with medium alone showed minimal background signal for Fc staining (left column). *Ptp10D* conditionally tagged brains incubated with Toll2-Fc-V5 proteins in medium have detectable signal for Fc staining in multiple brain regions (middle column). However, these signals are still present in *Ptp10D* mutant brains incubated with Toll2-Fc-V5 proteins (right column). These results suggest Toll2-Fc-V5 binds to other proteins expressed in the brain, masking the detection of its potential binding to Ptp10D.

As previous biochemical and structural studies have shown direct binding between Hbs/Sns and Kirre^75^, we also tested whether the other two CSP pairs we identified directly bind each other. However, we did not detect direct binding between Fili and Kek1, or between Toll2 and Ptp10D, as purified proteins or in tissue-based binding assays (Extended Data Fig. 6). Thus, the biochemical basis for the repulsive genetic interaction mediated by Fili–Kek1 and Toll2–Ptp10D remains an open question. Possibilities include requirements for unidentified co-factor(s), post-translational modifications, or specific physiological conditions like multimerization^81,82^ that were not recapitulated in our binding assays.

### Broad use of the three CSP pairs elsewhere

**Fig. 6 |:**
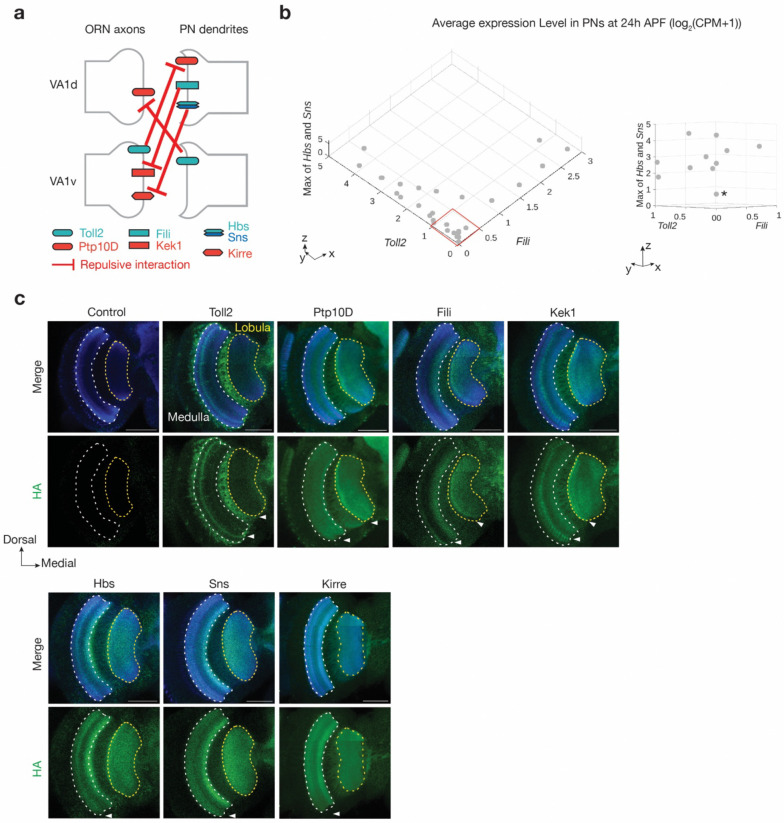
Three CSP pairs collectively cover the antennal lobe and differentially express in the optic lobe. **a,** Schematic summary of repulsive interactions of three CSP pairs in the VA1v and VA1d glomeruli. Hbs and Sns are combined as they perform similar functions. **b,** Single-cell RNA sequencing data showing the average expression level of non-cell-autonomous cues *Toll2*, *Fili,* and the maximum of *hbs* and *sns* throughout the adult antennal lobe at 24–30h APF. Right column is a zoom-in of the red box in the left column from a different angle, showing that PN types that express low levels of *Toll2* and *Fili* tend to express high levels of *hbs* or *sns* (* indicates a possible exception). Units for all axis: log_2_(CPM+1). CPM: counts per million reads. Data adapted from previous published studies^3,4^. **c,** Confocal images showing neuropil staining by N-cadherin antibody (blue) and HA staining (green) of tagged endogenous cell-surface proteins in the *Drosophila* optic lobe at 42–48 h APF. The medulla (white dotted lines) and lobula (yellow dotted lines) are outlined based on N-cadherin staining. Arrowheads indicate the layers with differential expression patterns. For example, Kek1 is highly expressed in a medulla layer that is low for Fili expression. Scale bars = 50 μm.

Our data suggest that the three repulsive CSP pairs could prevent mismatching between VA1d-ORNs with VA1v-PNs (Toll2 —| Ptp10d), VA1d-PNs with VA1v-ORNs (Toll2 —| Ptp10d), and VA1v-ORNs with VA1d-PNs (Fili —| Kek1; Hbs/Sns —| Kirre) (Fig. 6a). Thus, the three CSP pairs work in concert, with partial redundancy, to ensure robust repulsion between non-matching ORNs and PNs at the VA1d and VA1v glomeruli.

The antennal lobe has 50 ORN-PN synaptic partner pairs that need to be specified, potentially requiring many repulsive interaction pairs of CSPs. One way to alleviate this is to repeatedly use the same repulsive CSP pairs across the antennal lobe in a combinatorial fashion. Indeed, analysis of previously published single-cell transcriptomes during development^3^ revealed that mRNAs encoding all three CSPs implicated in sending the repulsive cues—Toll2, Fili, and the maximum of Hbs and Sns—are expressed in multiple PN types. Interestingly, most PN types expressed one or two but not all the three repulsive cues at high levels (Fig. 6b). For each PN type, this property reduces the errors of mismatching with non-partner ORN types (as all PN types express at least one repulsive cue at high levels) whereas makes room for the match with its partner ORN type (as no PN type expresses all three repulsive cues at high levels).

**Extended Data Fig. 7 |:**
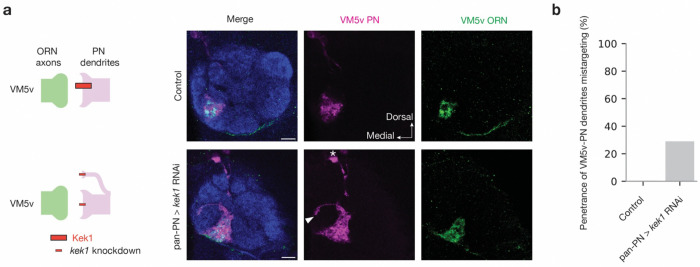
Kek1 knockdown in PNs cause ectopic targeting of VM5v-PN dendrites. **a,** Left column shows experimental schematic (red bar, high expression level of Kek1; smaller bar, knockdown). The right columns are confocal images showing adult antennal lobe neuropil staining by N-cadherin antibody (blue), VM5v-PN dendrites (magenta), and VM5v-ORN axons (green). Control VM5v-PN dendrites labeled by *GMR86C10-LexA* only innervate the VM5v glomerulus and fully overlap with VM5v-ORN axons labeled by *Or98a-mCD8GFP*. Some VA5v-PN dendrites mistarget to other glomerulus (arrowheads) in *kek1* RNAi driven by the pan-PN driver, phenocopying ORN knockdown of *Fili*^39^. * denotes PN cell body. **b,** Penetrance of the mistargeting phenotypes in **a**. For all genotypes, n ≥ 10. Scale bars = 10 μm.

**Extended Data Fig. 8 |:**
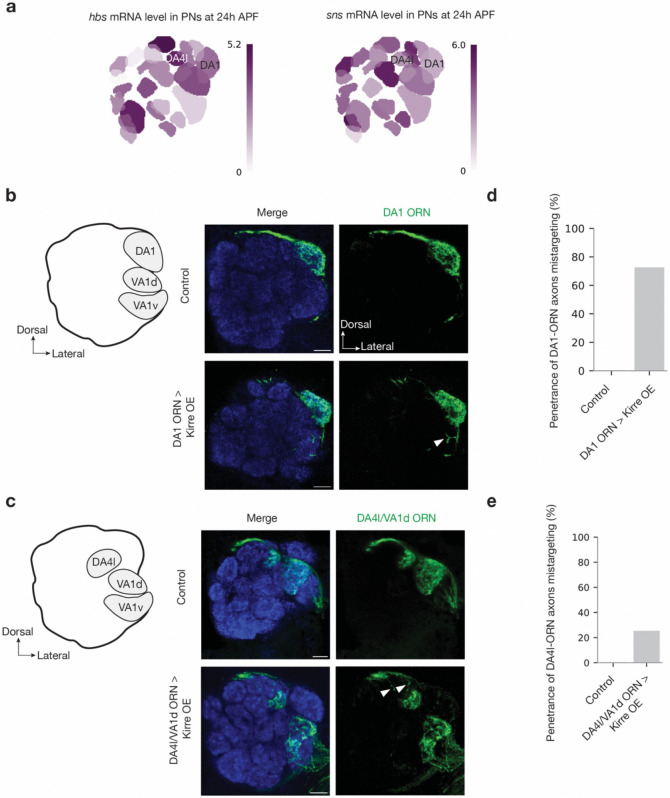
Kirre misexpression in DA1-ORNs and DA4l/VA1d-ORNs cause ectopic targeting of their axons. **a,** Single-cell RNA sequencing data showing the expression level of *hbs* and *sns* throughout the adult antennal lobe at 24–30 h APF. Heat map units: log_2_(CPM+1). CPM: counts per million reads. Data adapted from previous work^3,4^. **b, c,** Left column shows schematic of the adult antennal lobe with locations of three glomeruli highlighted in grey. Middle and right columns are confocal images showing adult antennal lobe neuropil staining by N-cadherin antibody (blue), DA1-ORN axons (green in **b**), and DA4l/VA1d-ORN axons (green in **c**). Some DA1-ORN axons or DA4l/VA1d-ORN axons mistarget to neighboring glomeruli (bottom rows) following Kirre overexpression (arrowheads), which is not observed in control (top rows). **d, e,** Penetrance of the mistargeting phenotypes in **b** (**d**) and **c** (**e**). For all genotypes, n ≥ 10. Scale bars = 10 μm.

Based on the expression pattern, we further investigated whether the repulsive interactions of these three CSP pairs play similar roles in other parts of the antennal lobe using loss- or gain-of-function experiments. A previous study showed that Fili acts in ORNs to regulate dendrite targeting of VM5-PNs^39^. We observed a similar mistargeting phenotype of VM5-PN dendrites when we knocked down Kek1 using a pan-PN driver (Extended Data Fig. 7), suggesting that Fili in ORNs and Kek1 in PNs are both required for proper targeting of VM5-PN dendrites. As Hbs is highly expressed in DA1-PNs and DA4l-PNs, we overexpressed Kirre in DA1-ORNs and DA4l/VA1d-ORNs, respectively. We observed ectopic targeting of each ORN group(s) to neighboring glomeruli (Extended Data Fig. 8), whose PNs expresses low levels of Hbs and Sns (Fig. 1o and Extended Data Fig. 2), suggesting that Hbs and Kirre mediate repulsive interactions in these PN-ORN pairs. In the companion manuscript, we show that all the three repulsive CSP pairs play a key role in preventing the mismatch of both VA1d-ORNs and DA1-ORNs with PNs of nearby glomeruli, as manipulating the expression of these CSP pairs is essential in the rewiring of VA1d- and DA1-ORNs to non-cognate PNs^51^. Altogether, these data support a model where these repulsive interactions are used broadly in the antennal lobe to ensure synaptic partner matching specificity.

Finally, to explore the possibility that these three CSP pairs work elsewhere in the fly brain to regulate connection specificity, we used HA staining to examine the expression patterns of the 7 CSPs (Fig. 1b) during the period when many circuits are establishing wiring specificity (42–48h APF). We found that all CSPs have broad but differential expression across the *Drosophila* brain (Supplementary Videos 1–7). For example, in the medulla and lobula of the optic lobe, all CSPs show differential expressions in specific neuropil layers (Fig. 6c). These data support the notion that combinatorial repulsive interactions serve as a generalizable mechanism in instructing wiring specificity of neural circuits.

## Discussion

Previous high-throughput extracellular interactome screenings *in vitro* have identified novel molecular pairs with direct interactions, including Dpr/DIP and Beat/Side families of immunoglobulin-containing CSPs whose *in vivo* functions in neuronal wiring have subsequently been validated^83,31,32,84,33^. Here, we took an alternative approach relying on transcriptome-informed *in vivo* genetic screens. This approach enabled us to identify known binding partners (Hbs/Sns–Kirre) as well as proteins that may not interact directly (Fili–Kek1 and Toll2–Ptp10D). Thus, transcriptome-informed *in vivo* screening could be complementary to *in vitro* biochemical approach to identify new CSP pairs that mediate trans-cellular interaction in neuronal wiring.

The inverse expression patterns of the three CSP pairs, their cell-type-specific loss-of-function phenotypes, and suppression assays strongly suggest that they mediate repulsion between non-partner PNs and ORNs. We note that orthologs of Hbs/Sns and Kirre in other species control synaptic cite choice in *C. elegans*^85,86^ and axon sorting in the mouse olfactory bulb^87^ via heterophilic and homophilic attraction, respectively. However, our results suggest they instruct synaptic partner matching in the fly olfactory system via heterophilic repulsion. These different mechanisms could potentially be mediated by engaging distinct intracellular signaling pathways in specific cellular context.

Repulsive interactions could combine with attractive interactions to enhance the selection process of synaptic partners. For neuron A to match its synaptic partner A’ but not non-partners (non-A’ neurons), one strategy is to express attractive CSP pairs in A and A’. However, as the CSP number on each synaptic partner increases, an attraction-only strategy can cause ambiguity (for example, to distinguish the matching of two vs. three attractive pairs) and the addition of repulsion can reduce the errors. Repulsion can also increase the searching efficiency by ruling out non-partners during the simultaneous searching process, as in the case of ORN-PN matching^45,46^. On the other hand, a repulsion-only strategy may have difficulty exploring a larger space due to excessive branch retraction. In the companion manuscript, we showed that only by simultaneously manipulating both attractive and repulsive CSPs in ORNs could we substantially switch their partner PNs^51^. Thus, attractive and repulsive interactions work in concert to ensure precise synaptic partner matching.

## Methods

### Generation of cDNA constructs and transgenic flies

Complementary DNA (cDNA) encoding for proteins used in this study were obtained from different resources. *Toll2* cDNA was amplified from the cDNA library of *w1118* pupal brain extracts using Q5 hot-start high-fidelity DNA polymerase (New England Biolabs) as previously described^46^; *Ptp10D* cDNA was amplified from clone RE52018 (DGRC Stock 9073; https://dgrc.bio.indiana.edu//stock/9073; RRID:DGRC_9073); *Fili* cDNA was amplified from *pUAST-attB-SP-V5-Fili-FLAG* plasmid^39^; *kek1* cDNA was amplified from clone GH23277 (DGRC Stock 1263019; https://dgrc.bio.indiana.edu//stock/1263019; RRID:DGRC_1263019); *kirre* cDNA was amplified from genomic DNA extraction of *UAS-kirre.C-HA* fly^88^ (RRID:BDSC_92196) using DNeasy blood and tissue kit (QIAGEN). Sequence-verified coding regions were assembled into *pUAST-attB-mtdT-3xHA*^89^, *pUAST-attB-SP-V5-Fili-FLAG*^46^ or *pJFRC19–13XLexAop2-IVS-myr::GFP* (Addgene Plasmid #26224) backbones using the NEBuilder HiFi DNA assembly master mix (New England Biolabs) to generate *pUAST-attB-Toll2-FLAG, pUAST-attB-Ptp10D-3xHA, pUAST-attB-Fili-FLAG, pUAST-attB-kek1–3xHA, pUASTattB-kirre-3xHA,* and *pJFRC19–13XLexAop2-IVS-Toll2* plasmids. Transgenic flies for overexpression experiments were generated by BestGene with microinjection of plasmids into the *VK5* site.

### Generation of conditional tags

Endogenous conditional tag flies were generated using CRISPR knock-in with modifications from a previous strategy^52^. To increase the efficiency of knock-in, we incorporated the short repair templates flanked by gRNA target sites^90^ and the gRNA into a single plasmid *TOPO-HR1-FRT-3xMyc-Stop-FRT-3xHA-Stop-loxP-mCherry-loxP-HR2-pU6-gRNA*. In the plasmid, *HR1* and *HR2* are the 150-bp genomic sequences of upstream and downstream of the target genes stop codon, respectively; *gRNA* sequences were designed by the flyCRISPR Target Finder tool that targeting stop codons of the genes, and were cloned into the backbone of *pU6-BbsI-chiRNA* vector^91,92^ to make the *pU6-gRNA*. The plasmids were synthesized by Synbio Technologies and were microinjected in house into *nos-Cas9* flies^93^. All *mCherry*+ progenies were individually balanced and the *loxP-*flanked *mCherry* cassettes were then removed by crossing each lines to balancer expressing Cre (Bloomington *Drosophila* Stock Center, RRID:BDSC 1092). To detect cell-type-specific expression level, we used *ey-Flp*^94^ for ORNs and *VT033006-GAL4;UAS-FLP*^95^ for PNs.

### Generation of the sparse driver and single-neuron genetic manipulations

The *FRT100-Stop-FRT100* element^45^ was cloned into the *78H05-p65AD* plasmid^96^ (in backbone *pBPp65ADZpUw*) to generate plasmid *78H05-FRT100-Stop-FRT100-p65AD* using the NEBuilder HiFi DNA assembly master mix (New England Biolabs). The plasmid was integrated into the *VK27* site. To perform the sparse genetic manipulations, flies including VA1d ORN sparse driver (*31F09-GAL4DBD*, *78H05-FRT100-Stop-FRT100-p65AD*), *hsFLP* (heat shock protein promoter-driven FLP), reporter (*UAS-mCD8-GFP*), and knockdown/overexpression transgenes were raised at 29°C. To induced sparse manipulation, the flies were collected at 0–6 hours after puparium formation and heat shocked for 1–2 hours in 37-degree water bath^97^.

### Immunostaining

Fly brains dissection, fixation, and immunostaining were done according to the published protocol^98^. For primary antibodies, we used rat anti-NCadherin (1:40; DN-Ex#8, Developmental Studies Hybridoma Bank), chicken anti-GFP (1:1000; GFP-1020, Aves Labs), rabbit anti-DsRed (1:500; 632496, Clontech), mouse anti-rat CD2 (1:200; OX-34, Bio-Rad), rabbit anti-HA (1:100, 3724S, Cell Signaling), mouse anti-HA (1:100, 2367S, Cell Signaling), and rabbit anti-Myc (1:250, 2278S, Cell Signaling). Donkey secondary antibodies conjugated to Alexa Fluor 405/488/568/647 or Cy3 (Jackson ImmunoResearch or Thermo Fisher) were used at 1:250. For the staining of conditional tag for Hbs and Fili in PNs, the routine protocol described above failed to detect Myc signal from the background, likely due to low expression of endogenous proteins *in vivo*. Alexa 488 Tyramide SuperBoost kit (Thermo Fisher) was used to amplify the immunostaining signal by following the manufacture’s protocol.

### Imaging, quantification, and statistical analysis

Images were obtained using laser scanning confocal microscopy (Zeiss LSM 780 or LSM 900). Fiji was used to adjust brightness and contrast for representative images. Penetrance of phenotypes represents the percentage of antennal lobe showing a given phenotype among the total antennal lobes (each animal has two antennal lobes) examined. To quantify the endogenous expression levels of the proteins, we manually outlined VA1d and VA1v glomeruli in Fiji based only on the NCad signal (i.e., blind to Myc signals), and use this as filter to calculate mean fluorescent density $\left(\bar{I}\right)$ in the VA1d and VA1v glomeruli (total fluorescence intensity divided by the volume). The preference index is calculated in Python by $\left({\overset{¯}{I}}_{\text{VA}1\text{d}}-{\overset{¯}{I}}_{\text{VA}1\text{v}})/({\overset{¯}{I}}_{\text{VA}1\text{d}}+{\overset{¯}{I}}_{\text{VA}1\text{v}}\right)$. To quantify the mistargeting ratio of VA1d-PNs or VA1d-ORNs in the trans-cellular assay, we defined PN dendritic or ORN axonal targeting area by smoothening (‘gaussian blur’ with radius = 1 pixels) and thresholding (based on the algorithm ‘Otsu’) the images in Fiji. Meanwhile, we manually outlined VA1d, VA1v, DA1, DA4l, and/or DC3 glomeruli in Fiji based only on the NCad signal (i.e., blind to PNs or ORNs signals), and use this as filter to calculate PN dendritic or ORN axonal targeting volume (V) in each glomerulus. The mistargeting ratio is calculated in Python by $V_{\mathrm{other}\;\mathrm{glomerulus}}/V_{VA1d}$.

### Binding assays

For binding assays involving purified proteins, we expressed and purified Fili, Kek1, Ptp10D, and Toll2 extracellular domains using the baculoviral expression system in *Trichoplusiani* High Five cells. Proteins were tagged with C-terminal hexahistidine tags for purification, and Avi-tags for biotinylation using BirA biotin ligase. Proteins were purified to homogeneity with Ni-NTA metal affinity and size-exclusion chromatography in 10 mM HEPES pH 7.2, 150 mM NaCl. Biotinylated Fili and Toll2 extracellular domains were captured on a Streptavidin sensor chip in a Biacore T200 system (Cytiva) running a buffer containing 10 mM HEPES pH 7.2, 150 mM NaCl and 0.05% Tween-20. We observed no binding responses for Kek1 and Ptp10D extracellular domains flowing on Fili and Toll2 channels, respectively.

The tissue-based binding assays were performed as previously described^83,99^. In brief, brains were dissected in the Schneider’s medium, then incubated with the conditioned medium of High Five cells expressing epitope-tagged extracellular domains of a specific protein for 18 hours at 4°C on a rotating platform. After the incubation, brains were washed with the Schneider’s medium and fixed with 4% paraformaldehyde in 1x PBS for 30 minutes, followed by the immunostaining protocol above using primary antibodies against the epitope tags.

## Extended Data

**Extended Data Table 1 |:** Information for genes and manipulations used in the genetic screen.

Gene	RNAi source	RNAi code	Manipulation	Penetrance
*CCKLR-17D3*	BDSC	67866	Mz19-PN	0/6
*CCKLR-17D3*	BDSC	67866	VA1d-ORN	4/5
*CG10474*	BDSC	51444	pan-PN	0/7
*CG14024*	VDRC	100991	pan-ORN	0/5*
*CG33143*	BDSC	28823	pan-PN	0/12*
*Con*	VDRC	17898	pan-ORN	7/7
*Con*	BDSC	28967	pan-ORN	4/5*
*Con*	BDSC	28967	pan-PN	3/7
*Cow*	BDSC	38235	pan-ORN	1/7*
*Cow*	BDSC	44530	pan-ORN	0/9*
*Cow*	BDSC	44530	pan-PN	1/8*
*cue*	VDRC	1043	pan-ORN	5/5*
*cue*	VDRC	104645	pan-ORN	0/5*
*cue*	BDSC	36875	pan-ORN	0/7
*Diap2*	VDRC	101187	pan-ORN	0/6*
*Dop1R1*	BDSC	62193	pan-PN	0/7
*ed*	BDSC	38209	pan-ORN	0/7*
*ed*	BDSC	38243	pan-ORN	0/4*
*ed*	BDSC	38243	pan-PN	3/7*
*Fas2*	BDSC	34084	Mz19-PN	0/7*
*Fas2*	BDSC	34084	pan-ORN	0/10*
*Fas2*	VDRC	103807	pan-ORN	0/7*
*Fas2*	BDSC	28990	pan-ORN	0/8*
*Fas3*	BDSC	77396	pan-PN	0/7
*Fas3*	BDSC	77396	pan-ORN	0/5*
*fra*	BDSC	40826	pan-PN	0/7
*fred*	BDSC	42621	DA1-ORN	0/6
*fred*	BDSC	42621	Mz19-PN	2/2
*fred*	BDSC	42621	pan-ORN	0/9*
*fred*	VDRC	46180	pan-ORN	1/6*
*fred*	VDRC	101138	pan-ORN	0/8*
*fred*	BDSC	42621	pan-PN	0/7*
*hbs*	VDRC	40898	pan-neuronal	8/12
*hbs*	BDSC	57003	pan-PN	3/6*
*Idit*	VDRC	101893	pan-ORN	0/6*
*Idit*	BDSC	28823	pan-ORN	0/12*
*Idit*	BDSC	28823	pan-PN	0/4*
*kek1*	VDRC	36252	pan-ORN	5/6*
*kek1*	BDSC	57000	pan-ORN	5/8
*kek1*	VDRC	101166	pan-ORN	1/8*
*kek5*	BDSC	40830	Mz19-PN	0/7
*kek5*	BDSC	40830	pan-ORN	7/7*
*kek5*	BDSC	40830	pan-PN	2/7*
*kek5*	BDSC	40830	VA1d-ORN	4/5
*kek6*	BDSC	61212	Mz19-PN	0/7
*kek6*	BDSC	61212	pan-ORN	7/7
*kek6*	VDRC	109681	VA1d-ORN	5/6
*kirre*	VDRC	109585	pan-ORN	9/12
*klg*	BDSC	28746	Mz19-PN	0/7
*klg*	BDSC	28746	pan-PN	0/7
*klg*	BDSC	28746	pan-ORN	6/9*
*klg*	VDRC	102502	pan-ORN	0/6*
*klg*	VDRC	108818	pan-ORN	0/7*
*Mcr*	BDSC	65896	pan-ORN	0/3*
*Mcr*	BDSC	65896	pan-PN	3/4*
*mgl*	BDSC	29324	pan-ORN	3/3*
*mgl*	VDRC	36389	pan-ORN	0/7*
*Nrt*	VDRC	8495	pan-ORN	0/8*
*Nrt*	BDSC	28742	pan-ORN	0/10*
*Nrt*	VDRC	106080	pan-ORN	0/6*
*Nrt*	BDSC	28742	pan-PN	0/7*
*PlexB*	BDSC	57813	Mz19-PN	3/8
*PlexB*	BDSC	57813	VA1d-ORN	0/5
*Ptp10D*	VDRC	8010	pan-ORN	4/8
*Ptp10D*	BDSC	39001	pan-ORN	8/13*
*Ptp10D*	BDSC	39001	pan-PN	7/7*
*Ptp99A*	BDSC	57299	DA1-ORN	7/7
*Ptp99A*	BDSC	57299	Mz19-PN	7/7
*Ptp99A*	BDSC	57299	pan-ORN	2/10*
*Ptp99A*	VDRC	103457	pan-ORN	0/8*
*Ptp99A*	VDRC	108505	pan-ORN	0/7*
*Ptp99A*	BDSC	57299	pan-PN	3/7
*rst* **	BDSC	28672	DA1-ORN	0/7
*rst* **	BDSC	28672	Mz19-PN	0/7
*rst* **	BDSC	28672	pan-PN	0/7
*sdk*	BDSC	33412	Mz19-PN	1/6
*sdk*	BDSC	33412	VA1d-ORN	0/5
*side-IV*	BDSC	82064	VA1d-ORN	10/10
*side-IV*	VDRC	102563	VA1d-ORN	5/7
*side-IV*	BDSC	82064	VA1d-PN	1/8
*side-IV*	VDRC	102563	VA1d-PN	2/12
*side-V*	BDSC	61953	Mz19-PN	2/9
*side-V*	BDSC	61953	VA1d-ORN	6/6
*slit*	VDRC	20210	pan-ORN	0/7*
*slit*	BDSC	31467	pan-ORN	3/10*
*slit*	BDSC	31468	pan-ORN	2/8*
*slit*	VDRC	108853	pan-ORN	0/7*
*slit*	BDSC	31467	pan-PN	0/7*
*slit*	BDSC	31468	pan-PN	0/7*
*sns*	VDRC	109442	pan-neuronal	14/20
*TkR86C*	VDRC	13392	pan-ORN	0/5*
*TkR86C*	VDRC	107090	pan-ORN	0/6*
*TkR86C*	BDSC	31884	pan-ORN	0/3*
*tnc*	BDSC	60058	pan-PN	7/7*
*Toll2*	VDRC	965	pan-ORN	8/8*
*Toll2*	BDSC	30498	pan-ORN	2/3*
*Toll2*	VDRC	36305	pan-ORN	2/3*
*Toll2*	VDRC	965	pan-PN	3/7

* indicates simultaneous knockdown of *Ten-m* using RNAi. Some of the screening experiments were done with the simultaneous knockdown of *Ten-m* to ‘sensitize’ the system because: (1) Ten-m has been shown to mediate the synaptic partner matching process^29^. (2) Ten-m is highly expressed in many of the glomeruli labeled in our screening. (3) Knocking down *Ten-m* leads to partial mismatching phenotype. In some of the ‘sensitized’ screening experiments, we followed up the initial phenotype with single manipulation that did not contain *Ten-m* knockdown. In general, we observed equal or higher penetrance when *Ten-m* was simultaneously knocked down. For example, in the *Toll2* RNAi experiments, the penetrance is 3/7 when using RNAi line 965 from VDRC alone and is 8/8 when using both line 965 and *Ten-m* RNAi.

** Rst (Roughest) binds with Hbs and Sns in vitro^75^ and *rst* is also expressed in PNs and ORNs based on the single-cell transcriptome data^3,4^. However, we did not observe any mistargeting phenotype in cell-type-specific *rst* knockdown experiments.

**Extended Data Table 2 |:** Summary of genotypes used in each experiment, arranged according to figure panels.

Figure and panel	description	Fly genotype		
		x chromosome	2nd chromosome	3rd chromosome
Figure 1				
c	Ptp10D in ORNs	*Ptp10D>HA>Myc (this study) / eyFLP*	*UAS>stop>mCD8-GFP (BDSC 30125) / +*	
	Ptp10D in PNs	*Ptp10D>HA>Myc*	*UAS-FLP (BDSC 4539) / +*	*VT033006- GAL4^95^ /* +
d	Toll2 in ORNs	*eyFLP^94^*	*Toll2>HA>Myc (this study) / UAS>stop>mCD8-GFP*	
	Toll2 in PNs		*Toll2>HA>Myc / UAS-FLP*	*VT033006-GAL4 / +*
e	Kek1 in ORNs	*eyFLP*	*kek1>HA>Myc (this study) / UAS>stop>mCD8-GFP*	
	Kek1 in PNs		*kek1>HA>Myc / UAS-FLP*	*VT033006-GAL4 / +*
f	Fili in ORNs	*eyFLP*	*Fili>HA>Myc (this study) / UAS>stop>mCD8-GFP*	
	Fili in PNs		*Fili>HA>Myc / UAS-FLP*	*VT033006-GAL4 / +*
g	Kirre in ORNs	*kirre>HA>Myc (this study) / eyFLP*	*UAS>stop>mCD8-GFP / +*	
	Kirre in PNs	*kirre>HA>Myc*	*UAS-FLP / +*	*VT033006-GAL4 / +*
h	Hbs in ORNs	*eyFLP*	*hbs>HA>Myc (this study) / UAS>stop>mCD8-GFP*	
	Hbs in PNs		*hbs>HA>Myc / UAS-FLP*	*VT033006-GAL4 / +*
i	Sns in ORNs	*eyFLP*	*sns>HA>Myc (this study) / UAS>stop>mCD8-GFP*	
	Sns in PNs		*sns>HA>Myc / UAS-FLP*	*VT033006-GAL4 / +*
Figure 2				
b	VA1d ORN Control^8^	*UAS-Dcr2* (BDSC 24646), *UAS-mCD8-GFP (BDSC 24648)*	*R20D10-QF2^8^, QUAS-mtdTomato-HA (BDSC 30004) / +*	*R78H05-p65.AD (BDSC 601815), R31F09-GAL4.DBD (BDSC 68759) / +*
c	*Ptp10D -/Y*	*Ptp10D[1]^56^* (BDSC 5810) */ Y*	*Mz19-Gal4 (BDSC 34497), UAS-mCD8-GFP^100^, Or88a-mtdt^69^ / +*	*Or47b-CD2 (BDSC 9915) / +*
d	VA1d ORN > *Ptp10D* RNAi	*UAS-Dcr2, UAS-mCD8-GFP*	*R20D10-QF2, QUAS-mtdTomato-HA (BDSC 30004) / +*	*R78H05-p65.AD, R31F09-GAL4.DBD / UAS-Ptp10D-RNAi (BDSC 39001)*
e	Single VA1d ORN > *Ptp10D* RNAi	*hsFLP^101^, UAS-mCD8-GFP*		*R78H05-FRT100-stop-FRT100-p65.AD (this study), R31F09-GAL4.DBD / UAS-Ptp10D-RNAi (BDSC 39001)*
f	*Toll2 −/−*		*18w[Delta7-35] / 18w[Delta7-35] ^61^ (BDSC 4372)*	*Or88a-mtdTomato^69^, Or47b-rCD2 / +*
g	VA1v PN > *Toll2* RNAi	*UAS-Dcr2, UAS-mCD8-GFP*	*Or88a-mtdTomato, Or47b-CD2 (BDSC 9916) / +*	*Dop1 R2- VP16.AD^70^ (gift from C. Desplan lab), VT033006-GAL4.DBD (BDSC 73333) / UAS-Toll2-RNAi (BDSC 30498)*
h	VA1d PN Control^8^	*UAS-Dcr2, UAS-mCD8-GFP*	*R73F07-p65.AD (BDSC 70805) / +*	*R26E12-GAL4.DBD (BDSC 70157)* / +
i	VA1d PN > *Ptp10D* RNAi	*UAS-Dcr2, UAS-mCD8-GFP*	*R73F07-p65.AD / +*	*R26E12-GAL4.DBD / UAS-Ptp10D-RNAi (BDSC 39001)*
j	VA1v ORN > *Toll2* RNAi	*UAS-Dcr2, UAS-mCD8-GFP*	*VT043692-p65.AD (BDSC 72503), R20D10-QF2, QUAS-mtdTomato-HA/ +*	*CG11317-GAL4.DBD^70^ (gift from C. Desplan lab) / UAS-Toll2-RNAi (BDSC 30498)*
Figure 3				
a	Control	*UAS-Dcr2, UAS-mCD8-GFP*	*R20D10-QF2, QUAS-mtdTomato-HA / +*	*R78H05-p65.AD, R31F09-GAL4.DBD / +*
b	VA1d ORN > Toll2 OE	*UAS-Dcr2, UAS-mCD8-GFP*	*R20D10-QF2, QUAS-mtdTomato-HA / P{GSV1}18w^EP-709^ (BDSC 43442)*	*R78H05-p65.AD, R31F09-GAL4.DBD / +*
c	*Ptp10D −/−*	*Ptp10D[1] / Y*	*R20D10-QF2, QUAS-mtdTomato-HA / +*	*R78H05-p65.AD, R31F09-GAL4.DBD / +*
d	*Ptp10D −/−, *VA1d ORN > Toll2 OE	*Ptp10D[1] / Y*	*R20D10-QF2, QUAS-mtdTomato-HA / P{GSV1}18w^EP-709^*	*R78H05-p65.AD, R31F09-GAL4.DBD / +*
f	Control	*UAS-Dcr2*	Mz19-AD*^G4HACK 8^, lexAop-rCD2::RFP-p10.UAS-mCD8::GFP-p10 (BDSC 67093) / +*	*R26E12-Gal4.DBD, R31F09-LexA.DBD^8^, R78H05-p65.AD / +*
g	VA1d ORN > Toll2 OE	*UAS-Dcr2*	Mz19-AD*^G4HACK^, lexAop-rCD2::RFP-p10.UAS-mCD8::GFP-p10 / P{GSV1}18w^EP-709^*	*R26E12-Gal4.DBD, R31F09-LexA.DBD, R78H05-p65.AD / +*
h	VA1d PN > *Ptp10D* RNAi	*UAS-Dcr2*	Mz19-AD*^G4HACK^, lexAop-rCD2::RFP-p10.UAS-mCD8::GFP-p10 / +*	*R26E12-Gal4.DBD, R31F09-LexA.DBD, R78H05-p65.AD / UAS-Ptp10D-RNAi (BDSC 39001)*
i	VA1d ORN > Toll2 OE, VA1d PN > *Ptp10D* RNAi	*UAS-Dcr2*	Mz19-AD*^G4HACK^, lexAop-rCD2::RFP-p10.UAS-mCD8::GFP-p10 / P{GSV1}18w^EP-709^*	*R26E12-Gal4.DBD, R31F09-LexA.DBD, R78H05-p65.AD / UAS-Ptp10D-RNAi (BDSC 39001)*
Figure 4				
b	VA1v ORN control	*UAS-Dcr2, UAS-mCD8-GFP*	*VT043692-p65.AD / +*	*CG11317-GAL4.DBD / +*
c	*kek1 −/−*		*kek1 mutant^74^ (NIG-fly M2L-1096) / kek1 mutant*	*Or47b-GAL4 (BDSC 9984), UAS-myr-GFP-p10 (gift from G. Rubin lab) / +*
d	VA1v ORN > *kek1* RNAi	*UAS-Dcr2, UAS-mCD8-GFP*	*VT043692-p65.AD / UAS-kek1-RNAi (VDRC 101166)*	*CG11317-GAL4.DBD / +*
f	VA1d ORN control	*UAS-Dcr2, UAS-mCD8-GFP*	*R20D10-QF2, QUAS-mtdTomato-HA / +*	*R78H05-p65.AD, R31F09-GAL4.DBD / +*
g	VA1d ORN > Kek1 OE	*UAS-Dcr2, UAS-mCD8-GFP*	*R20D10-QF2, QUAS-mtdTomato-HA / P{GSV1}kek1^EP-840^ (BDSC 43665)*	*R78H05-p65.AD, R31F09-GAL4.DBD / +*
h	Single VA1d ORN > Kek1 OE	*hsFLP, UAS-mCD8-GFP*	*P{GSV1}kek1^EP-840^ / +*	*R78H05-FRT100-stop-FRT100-p65.AD, R31F09-GAL4.DBD / +*
i	*Fili −/−*	*UAS-Dcr2, UAS-mCD8-GFP, QUAS-mtdTomato-HA^89^*	*Fili ex^18^39^ / FRTG13, Fili ex^18*	*R78H05-p65.AD, R31F09-GAL4.DBD* / +
j	*Fili −/+*, VA1d ORN > Kek1 OE	*UAS-Dcr2, UAS-mCD8-GFP, QUAS-mtdTomato-HA*	*Fili ex^18 / P(GSV1}kek1^EP-840^*	*R78H05-p65.AD, R31F09-GAL4.DBD / +*
k	*Fili −/−*, VA1d ORN > Kek1 OE	*UAS-Dcr2, UAS-mCD8-GFP, QUAS-mtdTomato-HA*	*Fili ex^18 / FRTG13, Fili ex^18, P(GSV1}kek1^EP-840^*	*R78H05-p65.AD, R31F09-GAL4.DBD / +*
Figure 5				
b	VA1v ORN control	*UAS-Dcr2, UAS-mCD8-GFP*	*VT043692-p65.AD /* +	*CG11317-GAL4.DBD / +*
c	*kirre -/Y*	*Df(1)duf_sps-1_^79^ (gift from M. Ruiz) / Y*		*Or47b-GAL4, UAS-myr-GFP-p10 / +*
d	VA1v ORN > *kirre* RNAi	*UAS-Dcr2, UAS-mCD8-GFP*	*VT043692-p65.AD / UAS-kirre-RNAi (VDRC 109585)*	*CG11317-GAL4.DBD / +*
e	*hbs −/+*		*FRT42D, hbs[66]^77^ (BDSC 27618) / +*	*Or47b-GAL4, UAS-myr-GFP-p10 / +*
f	VA1d/DA1 PN > *hbs* RNAi	*UAS-Dcr2, UAS-mCD8-GFP*	*Mz19-GAL4, UAS-mCD8-GFP, Or88a-mtdTomato / UAS-hbs-RNAi (VDRC 40898)*	*Or47b-CD2 / +*
g	*sns −/−*		*FRT42D, sns[XB3]^76^ / FRT42D, sns[XB3]*	*Or47b-GAL4, UAS-myr-GFP-p10 / +*
h	VA1d/DA1 PN > *sns* RNAi	*UAS-Dcr2, UAS-mCD8-GFP*	*Mz19-GAL4, UAS-mCD8-GFP, Or88a-mtdTomato / UAS-sns-RNAi (VDRC 109442)*	*Or47b-CD2 / +*
j	VA1d ORN control	*UAS-Dcr2, UAS-mCD8-GFP*	*R20D10-QF2, QUAS-mtdTomato-HA / +*	*R78H05-p65.AD, R31F09-GAL4.DBD / +*
k	VA1d ORN > kirre OE	*UAS-Dcr2, UAS-mCD8-GFP*	*R20D10-QF2, QUAS-mtdTomato-HA / +*	*R78H05-p65.AD, R31F09-GAL4.DBD / UAS-kirre-HA*
l	Single VA1d ORN > kirre OE	*hsFLP, UAS-mCD8-GFP*		*R78H05-FRT100-stop-FRT100-p65.AD, R31F09-GAL4.DBD / UAS-kirre-HA*
m	VA1d ORN > kirre OE, *sns −/−*	*UAS-myr-GFP-p10 (gift from G. Rubin lab)*	*SNS[XB3] / SNS[XB3]*	*R78H05-p65.AD, R31F09-GAL4.DBD / UAS-kirre-HA*
n	VA1d ORN > kirre OE, *hbs −/+*	*UAS-myr-GFP-p10*	*hbs[2593]^77^ / +*	*R78H05-p65.AD, R31F09-GAL4.DBD / UAS-kirre-HA*
o	VA1d ORN > kirre OE, *sns−/−, hbs−/+*	*UAS-myr-GFP-p10*	*SNS[XB3] / SNS[XB3], hbs[2593]^77^ (gift from M. Baylies)*	*R78H05-p65.AD, R31F09-GAL4.DBD / UAS-kirre-HA*
p	VA1d ORN > kirre OE, *kirre -/Y*	*Df(1)duf_sps-1_ / Y*	*UAS-myr-GFP-p10 (gift from G. Rubin lab)*	*R78H05-p65.AD, R31F09-GAL4.DBD / UAS-kirre-HA*
Figure 6				
c	homozygous conditional tagging flies (this study) for each gene			
Extended Data Figure 1				
a	VA1d ORN control		*Mz19-QF^29^, QUAS-mCD8-GFP^89^, Or88a-mtdTomato, Or47b-rCD2 /+*	
b	Pan-ORN > *Ptp10D* RNAi	*Pebbled-GAL4^102^, UAS-Dcr2*	*Mz19-QF, QUAS-mCD8-GFP, Or88a-mtdTomato, Or47b-rCD2 / +*	*UAS-Ptp10D-RNAi (BDSC 39001) / +*
c	Pan-PN > *Toll2* RNAi	*UAS-Dcr2*	*Mz19-QF, QUAS-mCD8-GFP, Or88a-mtdTomato, Or47b-rCD2 / +*	*VT033006-GAL4 / UAS-Toll2-RNAi (BDSC 30498)*
d	VA1v ORN control		*Mz19-QF, QUAS-mCD8-GFP, Or88a-mtdTomato, Or47b-rCD2 /+*	
e	Pan-ORN > *kek1* RNAi	*Pebbled-GAL4, UAS-Dcr2*	*Mz19-QF, QUAS-mCD8-GFP, Or88a-mtdTomato, Or47b-rCD2 / UAS-kek1 -RNAi (VDRC 101166)*	
f	Pan-ORN > *kirre* RNAi	*Pebbled-GAL4, UAS-Dcr2*	*Mz19-QF, QUAS-mCD8-GFP, Or88a-mtdTomato, Or47b-rCD2 / UAS-kirre-RNAi (VDRC 109585)*	
g	Pan-neuronal > *hbs* RNAi	*C155-GAL4^49^, UAS-Dcr2*	*Mz19-QF, QUAS-mCD8-GFP, Or88a-mtdTomato, Or47b-rCD2 / UAS-hbs-RNAi (VDRC 40898)*	
h	Pan-neuronal > *sns* RNAi	*C155-GAL4, UAS-Dcr2*	*Mz19-QF, QUAS-mCD8-GFP, Or88a-mtdTomato, Or47b-rCD2 / UAS-sns-RNAi (VDRC 109442)*	
Extended Data Figure 2	same as Figure 1			
Extended Data Figure 3				
a	VA1d ORN > *Ptp10D* RNAi	*UAS-Dcr2, UAS-mCD8-GFP, QUAS-mtdTomato-HA*	*R20D10-QF2 / +*	*R78H05-p65.AD, R31F09-GAL4.DBD / UAS-Ptp10D-RNAi (BDSC 39001)*
b	VA1d ORN > *Ptp10D* RNAi	*UAS-Dcr2*	*VT003280-LexA (BDSC 94678), lexAop-rCD2::RFP-p10.UAS-mCD8::GFP-p10 / +*	*R78H05-p65.AD, R31F09-GAL4.DBD / UAS-Ptp10D-RNAi (BDSC 39001)*
c	pan-ORN > *Toll2* RNAi	*Pebbled-GAL4, UAS-Dcr2*	*Mz19-QF, QUAS-mCD8-GFP, Or88a-mtdTomato, Or47b-rCD2 / +*	*UAS-Toll2-RNAi (BDSC 30498) / +*
d	VA1v-ORN > *Toll2* RNAi	*UAS-Dcr2, UAS-mCD8-GFP*	*VT043692-p65.AD, R20D10-QF2, QUAS-mtdTomato-HA/ +*	*CG11317-GAL4. DBD / UAS-Toll2-RNAi (BDSC 30498)*
e	VA1d ORN > Toll-2 OE (*UAS-Toll2*)	*UAS-Dcr2, UAS-mCD8-GFP*	*R20D10-QF2, QUAS-mtdTomato-HA / +*	*R78H05-p65.AD, R31F09-GAL4.DBD / UAS-Toll2-Flag*
f	VA1d ORN > Toll-2 OE		*R20D10-QF2, QUAS-mtdTomato-HA / P{GSV1}18w^EP-709^, Or43a-mCD8-GFP (BDSC 52625)*	*R78H05-p65.AD, R31F09-GAL4.DBD / +*
g	VA1v ORN > Ptp10D OE	*UAS-Dcr2, UAS-mCD8-GFP*	*VT043692-p65.AD / +*	*CG11317-GAL4.DBD / UAS-Ptp10D-HA*
Extended Data Figure 4				
d	VA1v ORN driver		*UAS-myr-GFP-p10 / VT043692-p65.AD*	*UAS-myr- GFP-p10 / CG11317-GAL4.DBD*
e	VA1v PN driver		*UAS-myr-GFP-p10 / +*	*UAS-myr- GFP-p10 / Dop1R2-VP16.AD, VT033006-GAL4.DBD*
g, h, i	VA1d-ORN sparse labeling control	*hsFLP, UAS-mCD8-GFP*		*R78H05-FRT100-stop-FRT100-p65.AD, R31F09-GAL4.DBD / UAS-Ptp10D-RNAi (BDSC 39001)*
Extended Data Figure 5				
	*sns−/−*		*SNS[S660] (BDSC 92179), UAS-gap-GFP / SNS[S660], UAS-gap-GFP*	*Or88a-mtdTomato, Or47b-rCD2 / Or88a-mtdTomato, Or47b-rCD2*
	*hbs−/+*		*FRT42D, hbs[66] (BDSC 27618) / +*	*Or88a-mtdTomato, Or47b-rCD2 / Or88a-mtdTomato, Or47b-rCD2*
	*sns−/−, hbs−/+*		*FRT42D, hbs[66] / SNS[XB3], hbs[2593]*	*Or88a-mtdTomato, Or47b-rCD2 / Or88a-mtdTomato, Or47b-rCD2*
	*kirre-/Y*	*Df(1)duf_sps-1_ / Y*		*Or88a-mtdTomato, Or47b-rCD2 / +*
Extended Data Figure 6	genotypes labeled on top of images			
Extended Data Figure 7				
a	VMSC control^39^		*R86C10-LexA^39^, LexAop-mtdTomato, Or98-mCD8-GFP, Or92a-rCD2 / +*	*VT033006-GAL4 / +*
b	pan-PN > *kek1* RNAi		*R86C10-LexA, LexAop-mtdTomato, Or98-mCD8-GFP, Or92a-rCD2 / UAS-kek1 -RNAi (VDRC 101166)*	
Extended Data Figure 8				
b	DA1-ORN control^46^	*UAS-Dcr2, UAS-mCD8-GFP*	*VT028327-p65.AD (BDSC 73064), Mz19-QF2* ^46^ *, QUAS-mtdTomato-HA / +*	*R22E04-GAL4.DBD (BDSC 69199) / +*
	DA1 ORN > Kirre OE	*UAS-Dcr2, UAS-mCD8-GFP*	*VT028327-p65.AD, Mz19-QF2, QUAS-mtdTomato-HA / +*	*R22E04-GAL4. DBD^46^ / P{EP}kirre[G876] (BDSC 26609)*
c	DA4I/VA1d ORN control^8^	*UAS-Dcr2, UAS-mCD8-GFP*	*VT023830-p65.AD (BDSC 72467) / +*	*R31F09-GAL4.DBD / +*
	DA4I/VA1d ORN > Kirre OE	*UAS-Dcr2, UAS-mCD8-GFP*	*VT023830-p65.AD / +*	*R31F09-GAL4.DBD / UAS-kirre-HA (this study)*

## Data Availability

All data are presented in the main figures, Extended Data Figures, and Extended Table 1. Any additional information is available upon requests to the corresponding author.
